# Transcriptional and Antagonistic Responses of Biocontrol Strain *Lysobacter enzymogenes* OH11 to the Plant Pathogenic Oomycete *Pythium aphanidermatum*

**DOI:** 10.3389/fmicb.2017.01025

**Published:** 2017-06-06

**Authors:** Yangyang Zhao, Guoliang Qian, Yuan Chen, Liangcheng Du, Fengquan Liu

**Affiliations:** ^1^Institute of Plant Protection, Jiangsu Academy of Agricultural SciencesNanjing, China; ^2^Key Laboratory of Integrated Management of Crop Diseases and Pests, College of Plant Protection, Nanjing Agricultural University, Ministry of EducationNanjing, China; ^3^Department of Chemistry, University of Nebraska-LincolnLincoln, NE, United States

**Keywords:** *Lysobacter enzymogenes*, *Pythium aphanidermatum*, transcriptome, interactions, HSAF, twitching motility

## Abstract

*Lysobacter enzymogenes* is a ubiquitous, beneficial, plant-associated bacterium emerging as a novel biological control agent. It has the potential to become a new source of antimicrobial secondary metabolites such as the Heat-Stable Antifungal Factor (HSAF), which is a broad-spectrum antimycotic with a novel mode of action. However, very little information about how *L. enzymogenes* detects and responds to fungi or oomycetes has been reported. An *in vitro* confrontation bioassay between the pathogenic oomycete *Pythium aphanidermatum* and the biocontrol bacterial strain *L. enzymogenes* OH11 was used to analyze the transcriptional changes in the bacteria that were induced by the oomycetes. Analysis was performed at three time points of the interaction, starting before inhibition zone formation until inhibition zone formation. A *L. enzymogenes* OH11 DNA microarray was constructed for the analysis. Microarray analysis indicated that a wide range of genes belonging to 14 diverse functions in *L. enzymogenes* were affected by *P. aphanidermatum* as critical antagonistic effects occurred. *L. enzymogenes* detected and responded to the presence of *P. aphanidermatum* early, but alteration of gene expression typically occurred after inhibition zone formation. The presence of *P. aphanidermatum* increased the twitching motility and HSAF production in *L. enzymogenes*. We also performed a contact interaction between *L. enzymogenes* and *P. aphanidermatum*, and found that HSAF played a critical role in the interaction. Our experiments demonstrated that *L. enzymogenes* displayed transcriptional and antagonistic responses to *P. aphanidermatum* in order to gain advantages in the competition with this oomycete. This study revealed new insights into the interactions between bacteria and oomycete.

## Introduction

Fungal-bacterial interactions are ubiquitous in complex ecological niches. They often influence each other's physiology and metabolism, and their interactions vary from synergism to mutualism to antagonism. For example, in a soil environment, which contains a wide range of bacteria and fungi in close proximity, the most documented interactions are those of the antagonistic rhizobacteria (e.g., *Pseudomonas* spp.), which play a role in preventing the establishment of plant pathogenic fungi in the rhizosphere. Other examples of synergism include bacteria helping symbiotic fungi to form tree mycorrhization or promoting disease development by pathogenic fungi in plants (Whipps, 2001; Frey-Klett et al., 2007; Barret et al., 2009).

The effects of bacteria on fungus on the molecular level have been widely studied. The influence on fungi of living bacteria at the gene expression level was reported by Deveau et al. (2007), who demonstrated that the mycorrhiza helper *Pseudomonas fluorescens* BBc6R8 induced growth and transcriptional changes in the ectomycorrhizal fungus *Laccaria bicolor* S238N. Candidate genes in the fungal defense response to biotic stress were revealed by studying the genes of the rice blast pathogen, *Magnaporthe oryzae*, when challenged with the bacterial antagonist *L. enzymogenes* (Mathioni et al., 2013). In addition, metabolites from bacteria can influence fungi at the transcriptional level. For example, Schoonbeek et al. (2002) discovered that 2,4-diacetylphloroglucinol, phenazine-1-carboxylic acid (PCA) and phenazine-1-carboxamide (PCN) broad-spectrum antibiotics produced by *Pseudomonas* spp., increased the expression of several ATP-binding cassette (ABC) transporter genes in *Botrytis cinerea*. A recent study reported that fungal innate immunity was induced when *Fusarium graminearum* was exposed to bacterial Microbe-Associated Molecular Patterns (MAMPs); this induction included increases in mitochondrial activity and iron sequestration, as well as the upregulation of genes that encode proteins involved in defense (Ipcho et al., 2016).

Though fungi respond to beneficial or antagonistic bacteria in many ways, accumulated evidence has revealed that fungal partners play important roles in influencing bacterial physiology, metabolism, and global gene expression. Romano and Kolter (2005) revealed that the yeast *Saccharomyces cerevisiae* had a positive effect on *Pseudomonas putida* bacterial physiology and survival, which was mediated by the yeast's ability to metabolize the available glucose, thereby altering the pH of the medium. Furthermore, Barret et al. (2009) showed that the plant pathogenic fungus *Gaeumannomyces graminis* significantly improved the growth of *P. fluorescens* Pf29Arp and triggered gene regulation in the early phases of their interaction. Others have also reported that the production of antibiotic compounds could be induced when antagonistic soil bacteria encountered other microorganisms (Becker et al., 1997). Antibiosis is probably the most widely studied interaction mechanism between fungi and bacteria (Frey-Klett et al., 2011). It has been documented that *Collimonas fungivorans* responds to the fungus *Aspergillus niger* by activating gene expression for fungal-derived compounds subsequently used in the production of a putative antifungal compound (Mela et al., 2011). The biocontrol species *Bacillus amyloliquefaciens* SQR9 regulated its gene expression and production of various antifungal compounds in response to different fungal pathogens (Li B. et al., 2014). For detrimental interactions, previous groups showed that medium pretreated with phytopathogenic oomycetes, *P. aphanidermatum*, decreased the expression of genes associated with ecological fitness in *P. fluorescens*, suggesting that a soluble fungal product may decrease the fitness of a bacterium in the environment (Fedi et al., 1997; Smith et al., 1999). It has been established that the biosynthesis of the diacetyl phloroglucinol antibiotic of *P. fluorescens* was inhibited by the production of a fusaric acid toxin by the filamentous fungus *Fusarium oxysporum* (Notz et al., 2002).

Despite the widespread occurrence of such bacterial-fungal interactions in myriad environments, it is not yet understood how biocontrol bacterial species detect and respond to other microbes at transcriptional level and by secondary metabolite production. The antagonistic activities of bacteria likely involve the production of an antibiotic compound, but it is not clear whether this provides an advantage to the bacteria over fungi in the competition for limited nutrients or enables mycophagous behavior (Mela et al., 2011).

*L. enzymogenes* belongs to the *Xanthomodaceae* family and is a ubiquitous environmental bacterium that is emerging as a potential biocontrol agent for the suppression of fungal and oomycete diseases. It exhibits several important traits, such as flagella-independent twitching motility, high G+C content, and dissimilarity to other taxonomically and ecologically related microbes (Christensen and Cook, 1978). The biocontrol ability of this bacterium against fungal and oomycete pathogens was originally attributed to the abundant production of lytic enzymes (such as chitinases, proteases, and glucanases) and the production of an antimicrobial secondary metabolite HSAF (Yu et al., 2007). The chemical structure, novel mode of action against filamentous fungi, and unique biosynthetic mechanism of HSAF have been explored in *L. enzymogenes* (Li et al., 2006, 2008; Li Y. Y. et al., 2014; Lou et al., 2011; Xu et al., 2015). There is no doubt that *L. enzymogenes* can inhibit fungi or oomycetes growth due to the actions of HSAF. However, very little information has been reported about how these newly identified potential biocontrol agents, such as *L. enzymogenes*, detect and respond to fungi, or oomycetes.

In this study, we investigated the interaction between the biocontrol agent *L. enzymogenes* and the plant pathogenic oomycete *P. aphanidermatum. P. aphanidermatum* is an important soil borne plant pathogen that causes damping-off of seedlings as well as root and crown rot in older plants, resulting in severe losses of many crops grown in closed soilless systems. *L. enzymogenes* has been shown to consistently suppress root and crown rot caused by *P. aphanidermatum* in bioassays on 2-week-old plants (Folman et al., 2003). *L. enzymogenes* strain 3.1T8 combined with chitosan can effectively control *P. aphanidermatum* in cucumber (Postma et al., 2009). Therefore, we investigated *Pythium-Lysobacter* interactions which potentially represent a novel microbial cross-talk system. The aim of the present work was to analyze the influence of *P. aphanidermatum* on the *L. enzymogenes* OH11 transcriptome and antibiotic production following non-contact commensal interactions. For this purpose, we used an *in vitro* confrontation assay to show that *P. aphanidermatum* exerted effects on the expression of a wide range of *Lysobacter* genes at three time points: before (24 h), during (48 h), and after (96 h) inhibition zones formation. A cDNA microarray was constructed and used to monitor *Lysobacter* transcriptional changes during its co-culture with *P. aphanidermatum*. The effects of *P. aphanidermatum* on HSAF production and the twitching motility of *L. enzymogenes* OH11 were also assessed. The results provide information to strengthen our understanding of the ecological fitness of *Lysobacter* in response to microbes of different niches.

## Materials and methods

### Strains and growth conditions used in this study

*L. enzymogenes* strains were cultured on 10% TSA (Tryptic Soy Agar) or in 10% TSB (Tryptic Soy Broth) at 28°C. *L. enzymogenes* strains used in this study include OH11, the wild-type (Jiang et al., 2005; Qian et al., 2009); 5E4, a *clp* mutant of *L. enzymogenes* C3 with inactive antifungal antagonism and biological control activities (Kobayashi et al., 2005); and K19, an HSAF-nonproducing mutant with a mutation in the ketosynthase domain of the PKS module of the *pks-nrps* gene, which is responsible for HSAF biosynthesis in *L. enzymogenes* C3 (Yu et al., 2007). The oomycete pathogen *P. aphanidermatum* was grown on 10% TSA at 26°C.

### *L. enzymogenes* and *P. aphanidermatum* confrontation assay

**Figure 1:**
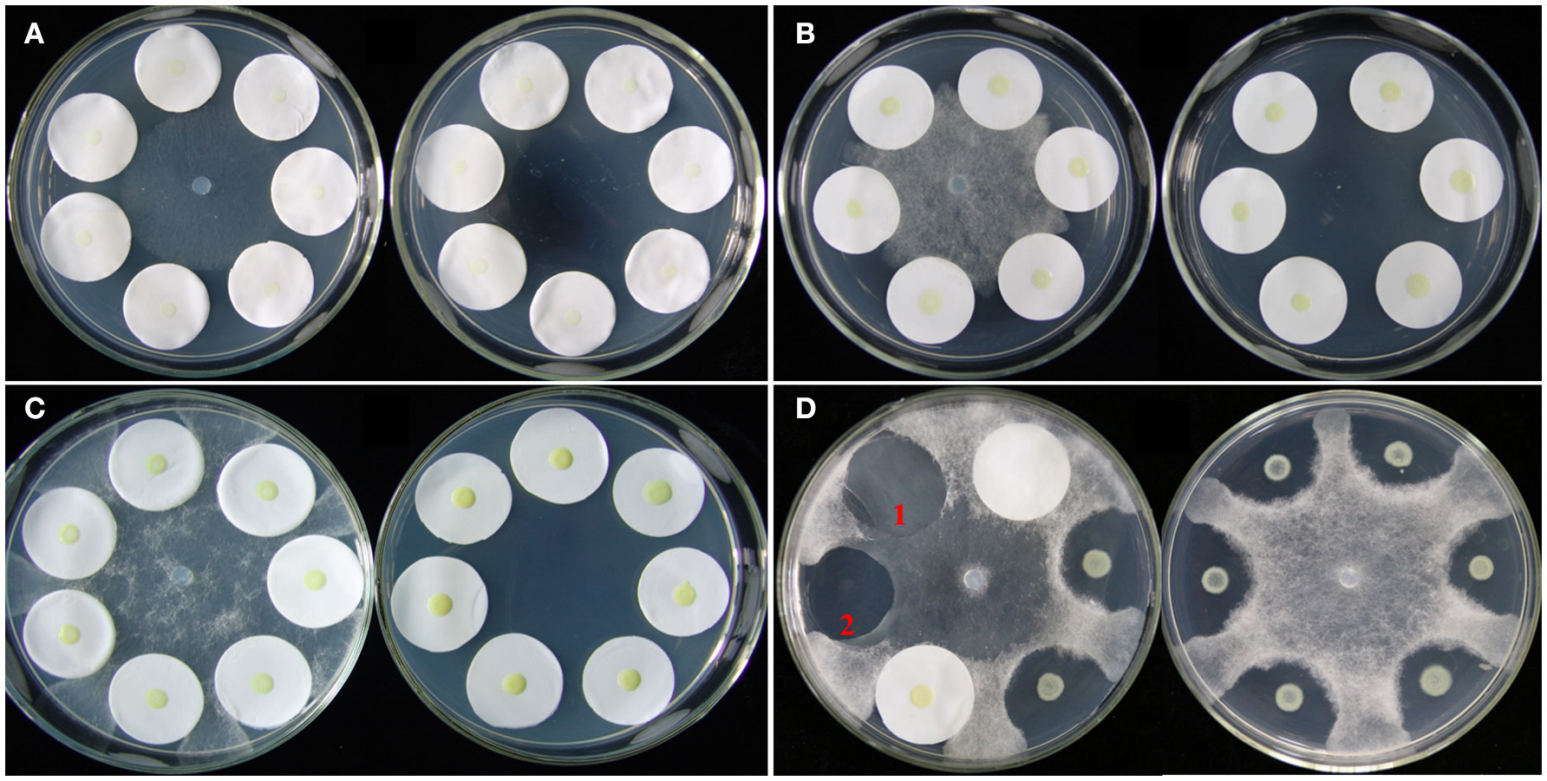
Co-cultivation of *L. enzymogenes* and *P. aphanidermatum* on agar plates. **(A)** Co-culture for 24 h (before inhibition zones formation); **(B)** Co-culture for 48 h (during inhibition zones formation); **(C)** Co-culture for 96 h (after inhibition zones formation). In each of these three sets, the left plate was *L. enzymogenes* and *P. aphanidermatum* co-cultured and the right plate was *L. enzymogenes* alone. **(D)** *Lysobacter-Pythium* assay with or without filter papers. The left plate shows that the filter paper alone does not block the oomycetes growth, and 1 indicates the hyphae grown area under the filter paper without OH11, while 2 indicates a clear inhibition zone under filter paper with OH11; the right plate represents *Lysobacter-Pythium* assay without filter papers.

A plate confrontation assay was developed to investigate the effects of *P. aphanidermatum* on *L. enzymogenes* (Figure 1). In the assay, a 5-mm diameter mycelial plug of *P. aphanidermatum* was cut out from the margins of a colony grown on 10% TSA medium and transferred to the center of a fresh 10% TSA plate. Cells of strain OH11 were washed twice and resuspended in sterile distilled water. Then, 5 μl droplets of bacterial suspension (OD_600nm_ = 1.0) were dropped on the center of a 2.5-cm diameter filter paper that surrounded the mycelial plug, and also dropped on the medium without filter papers as control. Six filter papers were placed in one dish, and two filter papers constituted one treatment. There were three technical replicates in a dish and three biological replicates (three different dishes). Under the same conditions, strain OH11 was grown alone as a control. Cultures were incubated at 28°C for 24 h (before inhibition zone formation), 48 h (during inhibition zone formation), and 96 h (after inhibition zone formation). At these three designated time points, the bacterial cells were collected from the filter papers and used for OD_600nm_ determination and RNA extraction. HSAF was extracted from the agar under the filter papers. The detailed methods are further described below. Bacterial colonies on the filter papers were washed with sterile distilled water; 2 ml of water was used to wash two filter papers, and then the OD_600nm_ of the suspension was detected by an Eppendorf BioPhotometer plus.

### HSAF extraction and detection

The extraction of HSAF from *L. enzymogenes* was performed as described previously (Yu et al., 2007; Lou et al., 2011), with some modifications. After removing the filter paper, the agar under the filter paper was collected and cut into small pieces. Then, these pieces were suspended in 5 ml of sterile water that had been acidified with 37% hydrochloric acid [0.4% (v/v)]. In the following steps, 5 ml of 100% ethyl acetate was added to the mixture for HSAF extraction. After gently shaking for 5 h, 2 ml of the ethyl acetate phase was collected and fully evaporated. Finally, the residues (containing HSAF) were dissolved in 100 μl of 100% methanol, the methanol extract was collected by centrifugation (10,000 × g at 4°C for 10 min), and the clear solution was directly used for high-performance liquid chromatography (HPLC, Agilent SB-C18 column, 5 μm, 4.6 × 250 mm) analysis. The mobile phase was 5 to 40% CH_3_CN in H_2_O from 0 to 10 min, 40 to 60% CH_3_CN in H_2_O from 10 to 15 min, 60 to 60% CH_3_CN in H_2_O from 15 to 20 min, 60 to 100% CH_3_CN in H_2_O from 20 to 22 min, 100% CH_3_CN from 22 to 24 min, 100 to 5% CH_3_CN in H_2_O from 24 to 26 min, and 5% CH_3_CN in H_2_O from 26 to 30 min (CH_3_CN and H_2_O containing 0.04% trifluoroacetic acid). The flow rate was 1.0 ml/min. The retention time of HSAF was 18.30 min. The yield of HSAF was displayed as the ratio of HSAF peak area and the OD_600nm_ of the bacterial suspension.

### Twitching motility assays

The twitching motility assays were performed as follows: first, a piece of 9 × 9 cm filter paper was placed in a dish, and a glass slide was put on the filter paper. Then, 2 ml of sterile distilled water was added to the filter to provide a moist environment, and then 1 ml of 5% TSA containing 1.7% agar was uniformly distributed on the glass slide. A 5-mm diameter mycelial plug of *P. aphanidermatum* from the margins of a colony grown on 10% TSA medium was transferred to a culture dish containing 5% TSA. Then, a cover glass with one edge dipped in a suspension of *L. enzymogenes* cells (washed twice with sterile distilled water, OD_600nm_ = 1.0) was gently laid on the medium without introducing air bubbles. *L. enzymogenes* derivatives were grown alone as a control. The cultures were incubated at 28°C for 24, 48, or 96 h. The twitching motility was observed by a microscope with 640-fold magnification. A classical phenomenon of twitching motility is that bacterial cells surge to the edge of a bacterial colony. Two replicates for each treatment were constructed, and the experiments were performed two times.

### RNA extraction, amplification and labeling

The bacterial cells of strain OH11 cultured with or without *P. aphanidermatum* were collected at the three designated time points noted above and used for RNA extraction with TRIzol reagent (Promega, USA) according to the manufacturer's instructions. The RNA was purified using the NucleoSpin® RNA clean-up kit (MACHEREY-NAGEL, Germany), and its purity was further assessed by formaldehyde agarose gel electrophoresis. RNA concentration was quantitatively determined by using a spectrophotometer (Agilent NanoDrop, USA). cDNA labeled with a fluorescent dye (Cy3-dCTP) was produced by Eberwine's linear RNA amplification method and subsequent enzymatic reactions, as described previously (Guo et al., 2005). Specifically, double-stranded cDNAs (containing the T7 RNA polymerase promoter sequence) were synthesized from 1 μg of total RNA using the CbcScript reverse transcriptase with cDNA synthesis system according to the manufacturer's protocol (CapitalBio, China) with the T7 Oligo (dT). After completion of the double-stranded cDNA (dsDNA) synthesis using DNA polymerase and RNase H, the dsDNA products were purified using a PCR NucleoSpin Extract II Kit (MN) and eluted with 30 μl of elution buffer. The eluted double-stranded cDNA products were evaporated in a vacuum to 16 μl and subjected to *in vitro* transcription reactions at 37°C for 4-14 h using T7 Enzyme Mix. The amplified cRNA was purified using the RNA Clean-up Kit (MN).

The Klenow enzyme labeling strategy was adopted after reverse transcription using CbcScript II reverse transcriptase. Briefly, 2 μg of amplified RNA was mixed with 4 μg of random nanomers, denatured at 65°C for 5 min, and cooled on ice. Then, 5 μl of 4 × first-strand buffer, 2 μl of 0.1 M DTT, and 1.5 μl of CbcScript II reverse transcriptase were added. The mixtures were incubated at 25°C for 10 min and then at 37°C for 90 min. The cDNA products were purified using a PCR NucleoSpin Extract II Kit (MN) and vacuum evaporated to a final volume of 14 μl. The cDNA was mixed with 4 μg of random nanomers, heated to 95°C for 3 min, and snap cooled on ice for 5 min. Then, 5 μl of Klenow buffer, dNTP, and Cy3-dCTP (GE Healthcare) were added to final concentrations of 240 μM dATP, 240 μM dGTP, 240 μM dTTP, 120 μM dCTP, and 40 μM Cy-dCTP. Finally, 1.2 μl of Klenow enzyme was added and the reactions were carried out at 37°C for 90 min. Labeled cDNA was purified with a PCR NucleoSpin Extract II Kit (MN) and resuspended in elution buffer.

### Microarray hybridization, scanning, and data analysis

Based on the sequence and annotation data for *L. enzymogenes* OH11 (data unpublished), microarrays were designed and produced by Roche NimbleGen (NimbleGen Systems of Iceland). The microarray slides contain specific oligonucleotides probes for 5,240 open reading frames of *L. enzymogenes* OH11. Briefly, the labeled samples were dried and dissolved in the hybridization solutions. The DNA suspended in hybridization solution was denatured at 95°C for 3 min prior to loading onto a microarray. Hybridization was performed at 42°C for 14 h with the NimbleGen hybridization system. The arrays were washed in the wash buffer I and II and III supplied by NimbleGen and dried in a NimbleGen microarray dryer. The arrays were scanned using an MS200 scanner (NimbleGen) with 2 μm resolution, and NimbleScan software (NimbleGen) was used to extract raw fluorescence intensity data from the scanned images. The probe expression data were normalized using quantile normalization (Bolstad et al., 2003), and the gene expression data were generated using the Robust Multichip Average (RMA) algorithm (Irizarry et al., 2003a,b). Microarray software (SAM, version 3.02) was used to identify differentially expressed genes. Genes were determined to be differentially expressed with false discovery rate (FDR) <5% and 2.0-fold change in the SAM output results.

### Real-time PCR

Quantitative real-time PCR (RT-qPCR) was carried out using the SYBR Premix EX Tag™ II kit (TaKaRa) in an ABI PRISM® 7500 Real-Time PCR System (Applied Biosystems); 16S rRNA was used as an endogenous control. The primer sequences used in this assay are listed in Table S2. RNA was extracted from various *L. enzymogenes* strains at different growth stages using the RNAiso plus reagent (Promega, USA) following the manufacturer's instructions. To remove genomic DNA, the eluted RNA samples were treated with RNase inhibitors and DNase I (TaKaRa). RNA integrity was confirmed by electrophoresis using 1.2% agarose gels. Then, 2 μg of each RNA sample was used to synthesize cDNA with a cDNA synthesis kit (TaKaRa). In this study, 10 differentially expressed genes from the microarray experiment were selected for validation with RT-qPCR at three interaction time points, with three replicates per treatment.

### Data analysis

All analyses were conducted using SPSS 14.0 (SPSS Inc., Chicago, IL, USA). The *t*-test (*P* = 0.05) was used to determine significant differences in bacterial growth and gene expression.

### Physical interactions of *L. enzymogenes* and *P. aphanidermatum*

The cultures of the *L. enzymogenes* wild-type strain OH11 and mutants were grown overnight in 10% TSB medium at 200 rpm at 28°C. The cultures were centrifuged at 6,000 rpm for 3 min. The supernatant was discarded, and the cultures were rinsed twice with sterile water. A spectrophotometer was used to measure OD_600nm_ of the bacteria, and the cultures were resuspended in sterile water to obtain a suspension at OD_600nm_ = 1.0. *P. aphanidermatum* grown on polyamide filter (1 cm-diameter) on 10% TSA were immersed in the bacterial suspension in a 6-well plate. The plate was placed in an incubator at 28°C until each time point was reached (10, 30 min, 2, 4, 6 h). After interacting for the corresponding time, the polyamide filter containing bacteria and oomycetes were fixed with 2% glutaraldehyde and then washed with sterile water. The images were taken with a Hitachi S-3000N scanning electron microscope.

## Results and discussion

### Effects of *P. aphanidermatum* on *L. enzymogenes* gene expression

**Figure 2:**
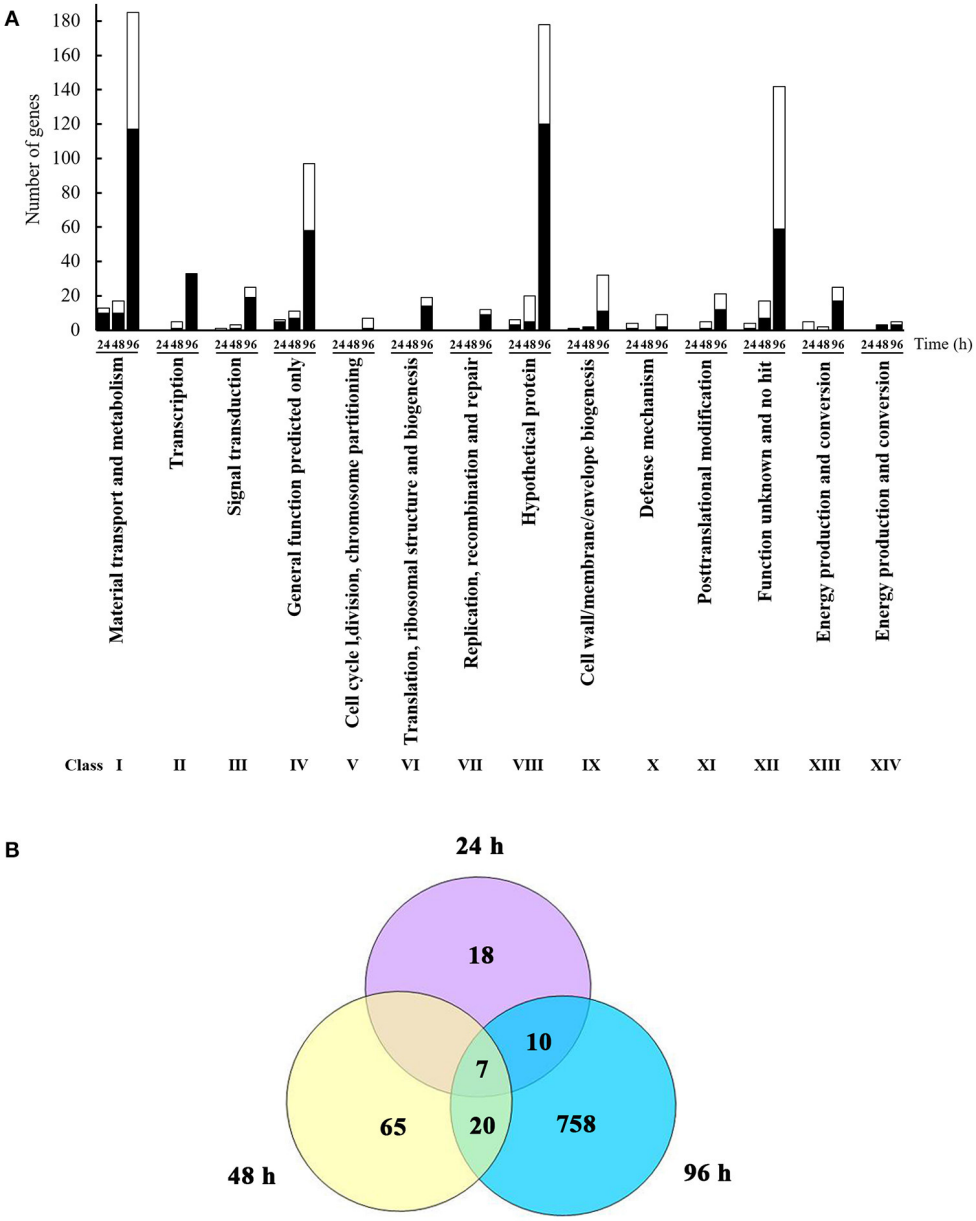
Identification and functional classification of differentially expressed genes of *L. enzymognes* influenced by the presence of *P. aphanidermatum* determined by DNA microarray. **(A)** The numbers of differentially expressed genes distributed in each functional class. Compared to *L. enzymogenes* OH11 monoculture, the black and white area in each bar showed the up and down expressed genes of *L. enzymogenes* in the presence of *P. aphanidermatum*, respectively. **(B)** The common differentially expressed genes at three or two interaction time points. Details were provided in Table 1 and Table S1.

*L. enzymogenes* has shown strong *in vitro* antibiosis against *P. aphanidermatum* (Folman et al., 2003). Folman et al. (2004) also showed that *L. enzymogenes* was a potential biocontrol agent of *P. aphanidermatum* in cucumbers. To understand the genetic basis for these and other responses of *L. enzymogenes* to *P. aphanidermatum* during non-contact confrontation, we performed transcriptome analysis using microarrays. The bacterial cells were collected at 24, 48, and 96 h after inoculation in the presence or absence of *P. aphanidermatum* and used for the extraction of RNA (Figure 1). As shown in Figure 2A and Table S1, the expression levels of 35, 92, and 795 genes were altered at the 24, 48, and 96 h time points, respectively. These differentially expressed genes belong to 14 functional groups, including material transport and metabolism; transcription; signal transduction; general function predicted only; cell cycle, division, chromosome partitioning; translation, ribosomal structure, and biogenesis; replication, recombination, and repair; hypothetical protein; cell wall/membrane/envelope biogenesis; defense mechanism; posttranslational modification; functions unknown and no hit, energy production and conversion protein; and cell motility. Specifically, compared to the *Lysobacter* monoculture, 22 and 13 genes were up- and down-regulated, respectively, at time point 24 h (before inhibition zone formation), with most of them belonging to the following functional groups: “material transport and metabolism” (13 genes; 37.14%); “general function predicted only” (6 genes; 17.14%); “energy production and conversion” (5 genes; 14.29%); and “hypothetical proteins” (6 genes; 17.14%). At time point 48 h (during inhibition zone formation), 35 and 57 genes were up- and down-regulated, respectively. These genes corresponded to the “material transport and metabolism” (17 genes; 18.48%); “General function predicted only” (11 genes; 11.96%); “hypothetical proteins” (20 genes; 21.74%); and “functions unknown and no hit proteins” (17 genes; 18.48%) groups. At 96 h (after inhibition zone formation), the number of differentially expressed genes was the largest of the three time points, with 480 and 315 genes up- and down-regulated, respectively. The top four groups corresponding to these differentially expressed genes were: “material transport and metabolism” (185 genes; 23.27%); “general function predicted only” (97 genes; 12.20%); “hypothetical proteins” (178 genes; 22.39%); and “function unknown and no hit proteins” (142 genes; 17.86%). In addition, a set of 10 differentially expressed genes among three time points were selected for validation with RT-qPCR amplification. As shown in Table S3, though there were some differences in the fold changes of several genes between RT-qPCR and microarray, the general trends were consistent between each other, suggesting that microarray data were valid.

As shown in Figure 2B and Table 1, only seven genes exhibited significantly changed expression levels at all three time points; they belong to energy production and conversion (2 genes), signal transduction mechanisms (1 gene), putative secreted protein (1 gene), hypothetical protein (2 genes), and no hit (1 gene) groups. Interestingly, 6 (*LysEGL005221-LysEGL005226*) of these seven genes are clustered together in the genome of strain OH11 (Figure S1). The transcriptional directions of five genes (*LysEGL005221-LysEGL005225*) were predicted to be consistent, and sequence overlap was also observed among *LysEGL005221, LysEGL005222*, and *LysEGL005223*. These results indicated that these five (*LysEGL005221-LysEGL005225*) genes may be co-transcribed. *LysEGL005221, LysEGL005222*, and *LysEGL005225* are hypothetical proteins, *LysEGL005223* and *LysEGL005224* are similar to cytochrome D ubiquinol oxidase, and *LysEGL005223* belongs to the universal stress protein family. Additionally, *LysEGL005226* was the only significantly down-regulated gene identified at all three time points. This gene is annotated encoding a universal stress protein whcih contains the UspA domain. The universal stress protein A (UspA) of *Escherichia coli* K-12 has been well characterized and is highly expressed in response to heat, substrate starvation, exposure to antimicrobial agents, and oxidative stress (Kvint et al., 2003). However, gene *LysEGL005226* was down-regulated in the presence of *P. aphanidermatum*, which may be due to the oomycetes inducing the bacteria to adopt a “relaxed” status in order to survive in the adverse environment. No differentially expressed genes were shared between the 24 and 48 h time points, whereas 10 differentially regulated genes overlapped between the 24 and 96 h time points, and 20 genes were present in both the 48 and 96 h time points.

**Table 1:** Common genes differentially expressed in *L. enzymogenes* when interaction with *P. aphanidermatum* at three or two time points.

**Functional class**	**Gene ID**	**Predicted product**	**24 h^a^**	**48 h^a^**	**96 h^a^**
(Material transport and metabolism)	LysEGL001192	3-isopropylmalate dehydratase, large subunit [*Stenotrophomonas maltophilia* R551-3]		2.1472	0.3341
	LysEGL004694	Homoserine dehydrogenase [*Stenotrophomonas maltophilia* R551-3]		2.1263	30.2053
	LysEGL005055	threonine dehydratase [*Xanthomonas oryzae* pv. *oryzae* MAFF 311018]		2.5976	5.0192
	LysEGL005056	2-isopropylmalate synthase [*Xanthomonas campestris* pv. *musacearum* NCPPB4381]		2.5174	2.2174
	LysEGL005057	glucose-methanol-choline oxidoreductase [*Shewanella baltica* OS185]		3.1632	3.4588
	LysEGL005058	probable 3-isopropylmalate dehydratase small subunit protein [*Xanthomonas albilineans*]		3.305	2.3477
	LysEGL005060	probable 3-isopropylmalate dehydrogenase protein [*Xanthomonas albilineans*]		3.4322	3.2653
	LysEGL003463	MprA [uncultured bacterium pTW2]		0.4839	0.1612
	LysEGL003465	MprA [uncultured bacterium pTW2]		0.3175	0.2088
	LysEGL003920	histidinol dehydrogenase [*Xanthomonas campestris* pv. *campestris* str. ATCC 33913]		0.4333	14.0833
	LysEGL000904	hypothetical glycosidase protein [*Xanthomonas albilineans*]	2.0746		0.4041
	LysEGL003025	Beta-N-acetylhexosaminidase [*Flavobacterium johnsoniae UW101*]	2.5844		0.1912
	LysEGL003267	beta-1,3-glucanase A [*Lysobacter enzymogenes*]	2.17		0.192
	LysEGL004434	beta-1,3-glucanase	2.6574		0.4161
	LysEGL004595	siroheme synthase [*Bordetella petrii* DSM 12804]		0.4034	9.7748
	LysEGL002652	sterol desaturase-like protein [*Lysobacter enzymogenes*]	0.4566		0.3319
	LysEGL002649 (*ox1*)	Ox1 [*Lysobacter enzymogenes*]	2.3545		0.1852
	LysEGL002651 (*pks-nrps*)	hybrid polyketide synthase and nonribosomal peptide synthetase [*Lysobacter enzymogenes*]	3.1719		0.2333
(Signal transduction)	LysEGL005226	Universal stress protein family [*Brevundimonas* sp. BAL3]	0.4774	0.4931	0.3729
(General function predicted only)	LysEGL000447	Putative secreted protein	2.0648	2.0113	0.2671
	LysEGL001346	R body protein RebB-like protein [*Burkholderia* sp. CCGE1003]		2.1517	0.2081
	LysEGL001347	R body protein RebB-like protein [*Burkholderia* sp. CCGE1003]		2.2947	0.1672
	LysEGL002784	lipase family protein [*Cellvibrio japonicus* Ueda107]		2.2209	0.4031
(Hypothetical protein)	LysEGL005221	hypothetical protein Avin_19860 [*Azotobacter vinelandii* DJ]	0.331	0.415	0.265
	LysEGL005222	hypothetical protein Bpet0458 [*Bordetella petrii* DSM 12804]	0.3243	0.4165	0.1208
	LysEGL000784	conserved hypothetical protein [*Xanthomonas oryzae* pv. *oryzae* KACC10331]		0.3873	4.7785
	LysEGL003151	hypothetical protein Swit_3175 [*Sphingomonas wittichii* RW1]		0.2422	2.7647
	LysEGL003233	hypothetical protein Bphyt_1886 [*Burkholderia phytofirmans* PsJN]		5.7147	2.1411
(Cell wall/membrane/envelope biogenesis)	LysEGL004924	OmpW family outer membrane protein [*Xanthomonas campestris* pv. *vesicatoria* str. 85-10]	2.2362		0.2816
(Defense mechanisms)	LysEGL003010	polysaccharide biosynthesis protein [*Prevotella melaninogenica* ATCC 25845]	2.4973		0.2803
(Function unknown and no hit)	LysEGL004918		0.3603		0.3502
	LysEGL005225		0.4782	0.385	0.0989
	LysEGL000217			2.3147	0.4931
	LysEGL002605			2.6681	0.3409
	LysEGL002606			2.3923	0.34
(Energy production and conversion)	LysEGL005223	Cytochrome d ubiquinol oxidase, subunit II [*Rhodoferax ferrireducens* T118]	0.3095	0.3939	0.1493
	LysEGL005224	Cytochrome D ubiquinol oxidase, subunit I [*Legionella pneumophila* str. Corby]	0.3998	0.4066	0.1112

a *Red colors show up-regulated genes in L. enzymogenes caused by the presence of Pythium aphanidermatum, while green colors indicate down-regulated genes (Fold change ≥2 or ≤ 0.5)*.

### HSAF biosynthetic genes alteration and HSAF production

The antagonistic activity of *L. enzymogenes* against fungi or oomycetes was due to HSAF which is produced by *L. enzymogenes* and exhibits strong antimycotic activity against a wide range of fungi and oomycetes (Folman et al., 2004; Yu et al., 2007; Li et al., 2008). In the confrontation assay, *L. enzymogenes* inhibited the growth of *P. aphanidermatum*, and formed a clear inhibition zone on the nutrient-limiting medium (10% TSA; Figure 1D).

HSAF-deficient mutants lack antagonism activity against fungi and oomycetes; therefore, HSAF is a key factor for *L. enzymogenes* antagonism of fungi and oomycetes (Li et al., 2008). The HSAF biosynthetic gene cluster contains a *pks-nrps* gene encoding a single-module polyketide synthase/nonribosomal peptide synthetase and four genes (*ox1-ox4*) which encode a cascade of NADP/FAD-dependent oxidoreductases, all of which are involved in HSAF biosynthesis (Li et al., 2008; Lou et al., 2011). In this study, we showed that *pks-nrps* and *ox1* genes were up-regulated in *L. enzymogenes* after co-culture with *P. aphanidermatum* for 24 h, whereas the expression of all five genes decreased at the 48 and 96 h time points (Tables S1, S3). The results possibly indicate that at 24 h, before inhibition zone formation, *L. enzymogenes* sensed the presence of *P. aphanidermatum* and increased the expression of HSAF biosynthetic genes to produce and accumulate HSAF to inhibit the growth of the oomycetes. At the 48 and 96 h time points, inhibition zones were formed and stabilized, *P. aphanidermatum* may have sensed that danger from the bacterial enemy had diminished and subsequently inhibited the expression of genes related to HSAF biosynthesis.

**Figure 3:**
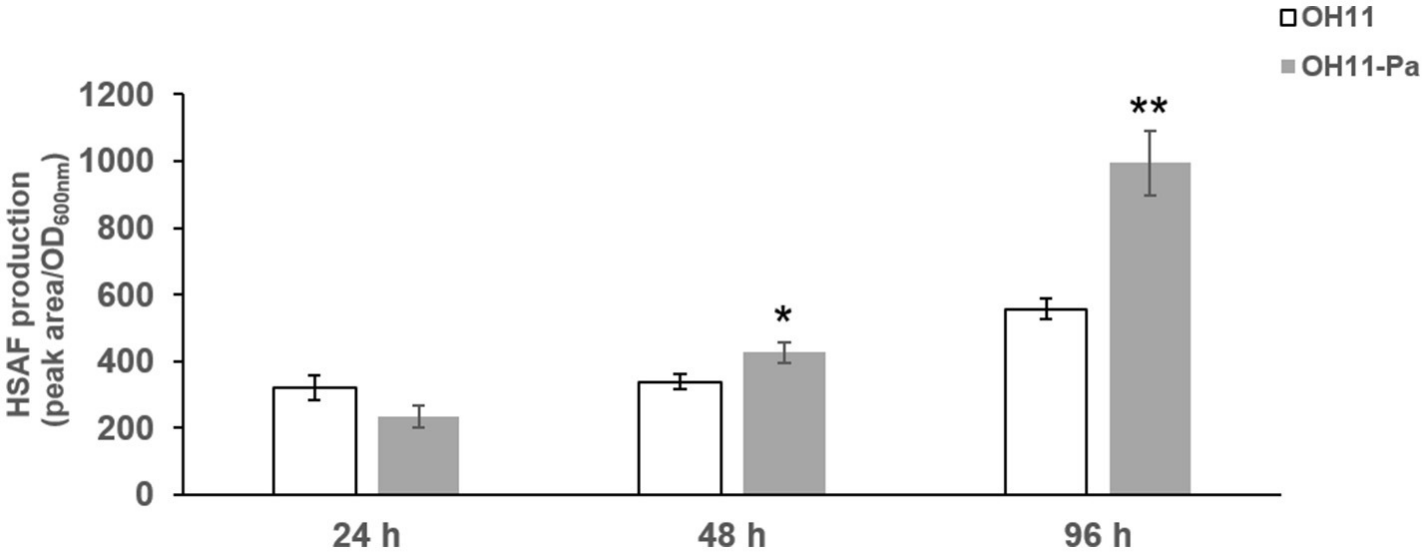
Determination of HSAF yield produced by *L. enzymogenes* in the presence or absence of *P. aphanidermatum* at 24, 48, 96 h. OH11, monoculture of the wild-type strain of *L. enzymogenes*. OH11-Pa, OH11 was co-cultured with *P. aphanidermatum*, as shown in Figure 1. HSAF production of OH11 was illustrated in Peak area/OD_600nm_ as means of three biological replicates, each containing two or three technical replicates. Peak area indicated the area of HSAF determined by HPLC method, while OD_600nm_ represents the growth status of tested strains at the time points used for the extraction of HSAF. Vertical bars indicated standard errors of three biological replicates. Significant difference in HSAF production between OH11 monoculture and co-cultured with *P. aphanidermatum* according to a *t*-test (^*^p < 0.05, ^**^p < 0.01).

To further investigate the effect of *P. aphanidermatum* on *L. enzymogenes*, we studied the variation of HSAF production in the bacteria at the three interaction time points. To calibrate the cell density of different cultures with HSAF yield, we used the ratio of peak area/OD_600nm_ to quantitatively evaluate the HSAF production in *L. enzymogenes*. Here, the peak area of HSAF was determined by HPLC. We used the OD_600nm_ to represent the cell density of the tested strains at the corresponding time point. We observed that the HSAF yield was increased significantly after 48 and 96 h of interaction whereas not changed at 24 h in the presence of *P. aphanidermatum* compared to *L. enzymogenes* monoculture (Figure 3). Antibiosis may be a common defensive or offensive strategy in microbial interactions (Garbeva et al., 2011). The mechanism used by *L. enzymogenes* regulating HSAF production in the presence of oomycetes is not clear.

### OH11-responsive genes involved in material transport and metabolism

At all three time points in the non-contact interaction experiments, a large percentage of differentially expressed *L. enzymogenes* genes were involved in material transport and metabolism. The predicted functions of these genes were linked to the transport and metabolism of amino acids, nucleotides, carbohydrates, coenzymes, lipids and ions. Of these, most genes (117 genes, 63.24%) were up-regulated (Table S1). It must be noted that the genes corresponding to nucleotide, coenzyme, lipid, and ion transport and metabolism were mostly observed to be differentially expressed at the late-interaction stage (96 h). Expression levels of ten tonB-dependent receptors related to iron transport and metabolism were significantly changed at 96 h.

TonB-dependent receptors (TBDRs) are bacterial outer membrane proteins in gram-negative bacteria responsible for the uptake of scarce resources from competitive environments. TBDRs were characterized as importers of Fe3^+^-siderophore complexes (Hantke, 1983), and some TBDRs were shown to be involved in the import of non-Fe compounds, such as vitamin B12, sugars, and non-Fe cations (Schauer et al., 2008). The possible cross-talk event between *L. enzymogenes* and *P. aphanidermatum* resulted in the differential expression of ten genes related to TBDRs, which were all significantly down-regulated at 96 h, although their expression levels were unchanged at both 24 and 48 h. It has been reported that a TBDR played a key role in regulating antibiotic (HSAF) biosynthesis in *L. enzymogenes* (Wang et al., 2016), but the transcription level of this TBDR encoding gene was not changed in the *Lysbacter-Pythium* interaction. These results suggested that silencing *TBDR* gene expression during the late-interaction stage might be a strategy used by *P. aphanidermatum* to obtain an advantage in limited nutrition conditions. The function of these ten regulated *TBDRs* genes in *Lysbacter-Pythium* interaction will be further studied.

### OH11-responsive genes associated with signal transduction

At the 24 h time point, only one locus associated with signal transduction, encoding a universal stress protein, was differentially expressed (down-regulated); 28 signal transduction genes were found to be differentially expressed at the 48 and 96 h time points. Of these, 25 genes were up-regulated at the 96 h time point, and eight genes belonged to the two-component signal transduction systems (Table S1).

Two-component signal transduction systems (TCSTSs), composed of a membrane-bound histidine kinase sensor (HK), and a response regulator (RR), are the main sense-response mechanisms that regulate the wide range of physiological pathways (Stock et al., 2000) that respond to environmental stimuli in different bacterial species, such as *Xanthomonas campestris* pv. *campestris* (Qian et al., 2008; Wang et al., 2010), *Pseudomonas syringae* (Lavin et al., 2007), and *Erwinia amylovora* (Zhao et al., 2009). To date, the diverse functions of TCSTSs in bacteria are linked to cell-cell signaling, chemotaxis, sporulation, osmolarity, nutrient assimilation, antibiotics production, and virulence (Stock and Guhaniyogi, 2006). The ability of *L. enzymogenes* to attach to and infect fungal hypha was reported to be dependent on the production of type IV pilus (T4P), which is a thin, hair-like appendage formed from pilin, or PilA, subunits (Patel et al., 2011). Two response regulator PilG and PilR belonging to TCSTSs involved in T4P biosynthesis have been shown that they activated twitching motility and downregulated HSAF production in *L. enzymogenes* (Zhou et al., 2015; Chen et al., 2017). However, the expression of *pilG* and *pilR* were not regulated in this interaction. The results suggested that *pilG* and *pilR* didn't play important roles in bacteria-oomycetes interaction, and the eight altered TCSTSs related genes above may involve in the interaction but not through affecting HSAF production and twitching motility in *L. enzymogenes*.

### Alteration of twitching motility

**Figure 4:**
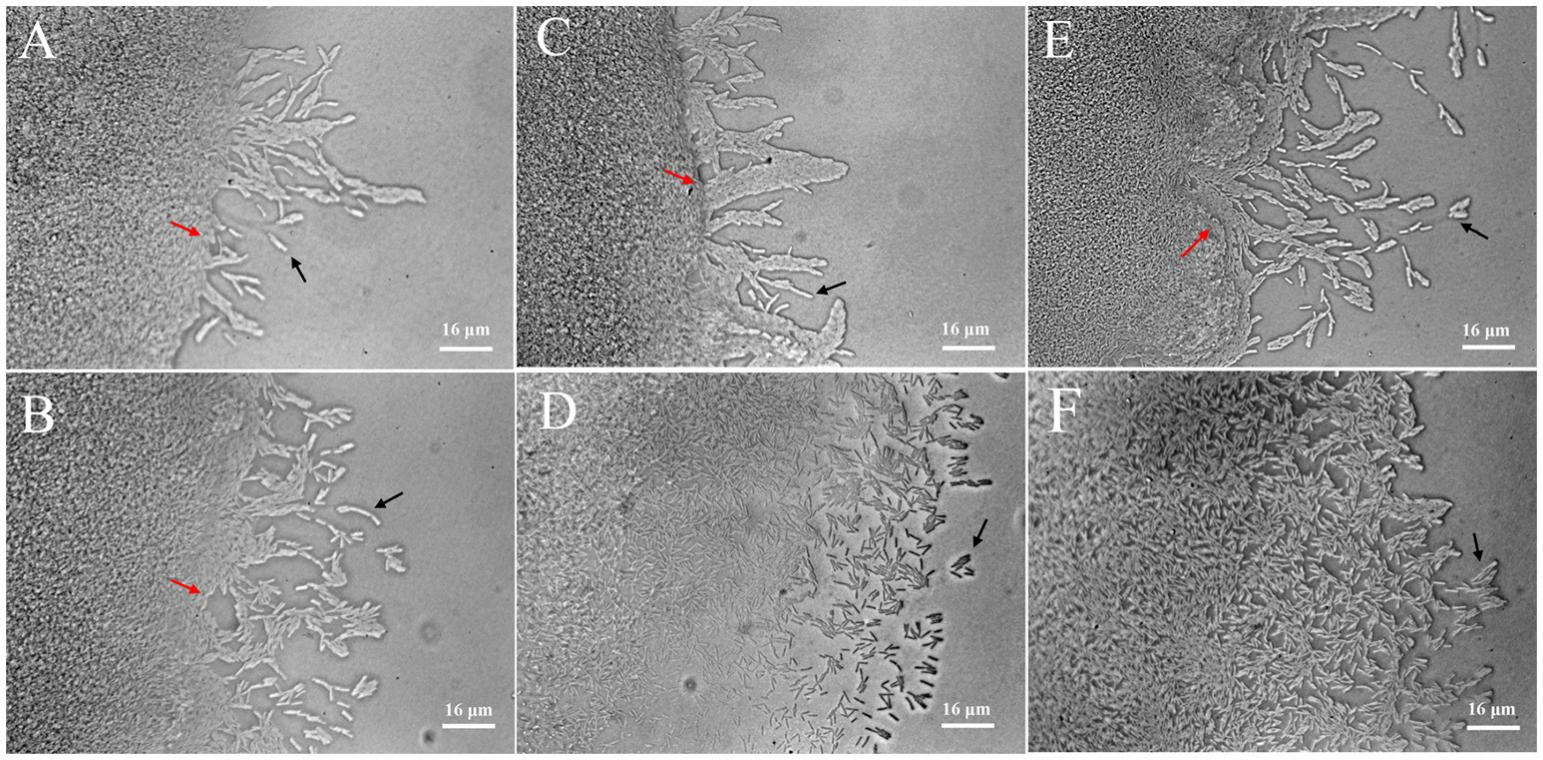
Detection of twitching motility of *L. enzymogenes* OH11 in the presence or absence of *P. aphanidermatum*. *L. enzymogenes* monoculture (up), co-culture with *P. aphanidermatum* (down). **(A,B)**, co-culture for 24 h; **(C,D)**, co-culture for 48 h; **(E,F)**, co-culture for 96 h. When *L. enzymogenes* monoculture (up), there were a small number of cells moving out, whereas more motile cells at the leading edge of the moving zone when co-culture with *P. aphanidermatum* (down). Red arrows indicate the colony edge, and black arrows indicate the motile cells. In **(E,F)**, colony edge can't be seen clearly because of too many cells moving away from the edge. The areas photographed represent the outermost end of cell growth (magnification, ca. × 640).

Twitching motility is a typical phenotypic characteristic for the flagella-less *L. enzymogenes* species (Mattick, 2002; Sullivan et al., 2003). Twitching motility occurs by the extension, tethering, and retraction of polar type IV pili (T4P), which is controlled by a large number of genes and a range of signal transduction systems, including two-component sensor-regulators and a complex chemosensory system (Mattick, 2002). However, we find that T4P-related genes were not regulated in *L. enzymogenes* OH11 in the presence of *P. aphanidermatum*. Although the importance of twitching motility for *L. enzymogenes* biological control activity has not been investigated, twitching motility is considered to be crucial for the spread and colonization of bacteria inside host xylem vessels (Burdman et al., 2011). Therefore, we observed the changes in twitching motility of *L. enzymogenes* when co-cultured with *P. aphanidermatum*. When strain OH11 grew alone, individual or small clusters of cells separated from the mass of cells at the colony margin. After 24 h of interaction, the increase in bacteria motility in the presence of *P. aphanidermatum* was not obvious. Whereas, scattered cells moved out of the leading edge of the colony when co-cultured with *P. aphanidermatum* for 48 h, after 96 h interaction, an abundance of bacteria aggregated toward the mycelia (Figure 4).

The twitching motility of *L. enzymogenes* was strengthened when co-cultured with *P. aphanidermatum*, which suggested that more *L. enzymogenes* cells might be trying to move toward the mycelia to effectively inhibit the oomycetes or colonize the mycelia. This may be an antagonistic response employed by a biocontrol agent in adaptation to the threat from the external environment.

### Physical interactions of *Lysobacter-Pythium*

**Figure 5:**
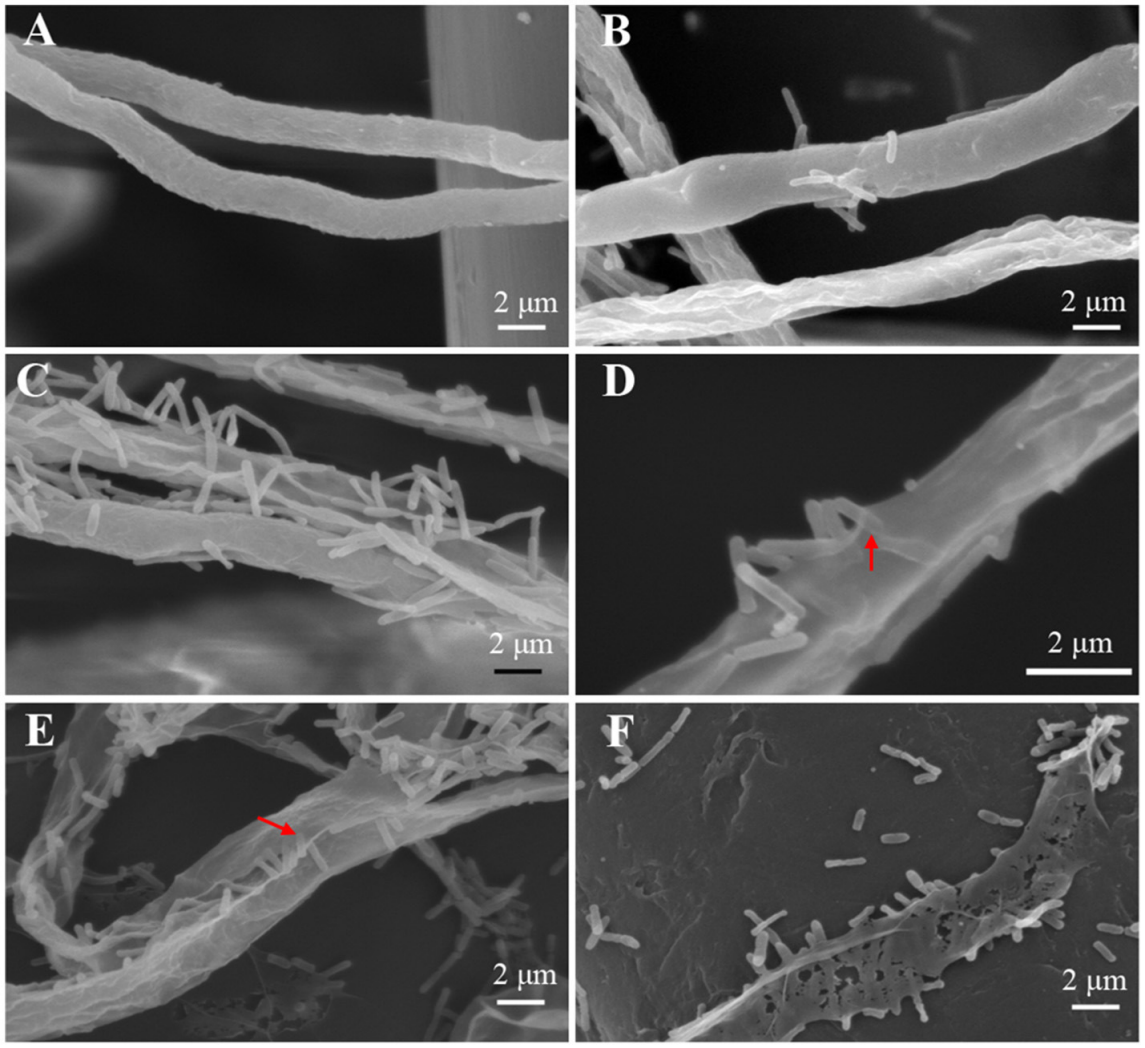
SEM examination of the *Pythium* hyphae and *Lysobacter* cells during oomycete-bacterium physical interactions. **(A)** Control, *P. aphanidermatum* alone; **(B)** Interaction for 10 min, showing that *Lysobacter* cells started to attach to the hyphae; **(C)** Interaction for 30 min, showing that more *Lysobacter* cells attached to the hyphae; **(D)** Interaction for 2 h, showing that *Lysobacter* cells invaded into the mycelium, red arrow indicates the invading bacterium; **(E)** Interaction for 4 h, showing that more *Lysobacter* cells into the *Pythium* hyphae, red arrow indicates bacteria into hyphae; **(F)** Interaction for 6 h, showing that *Pythium* hyphae were almost totally disintegrated.

**Figure 6:**
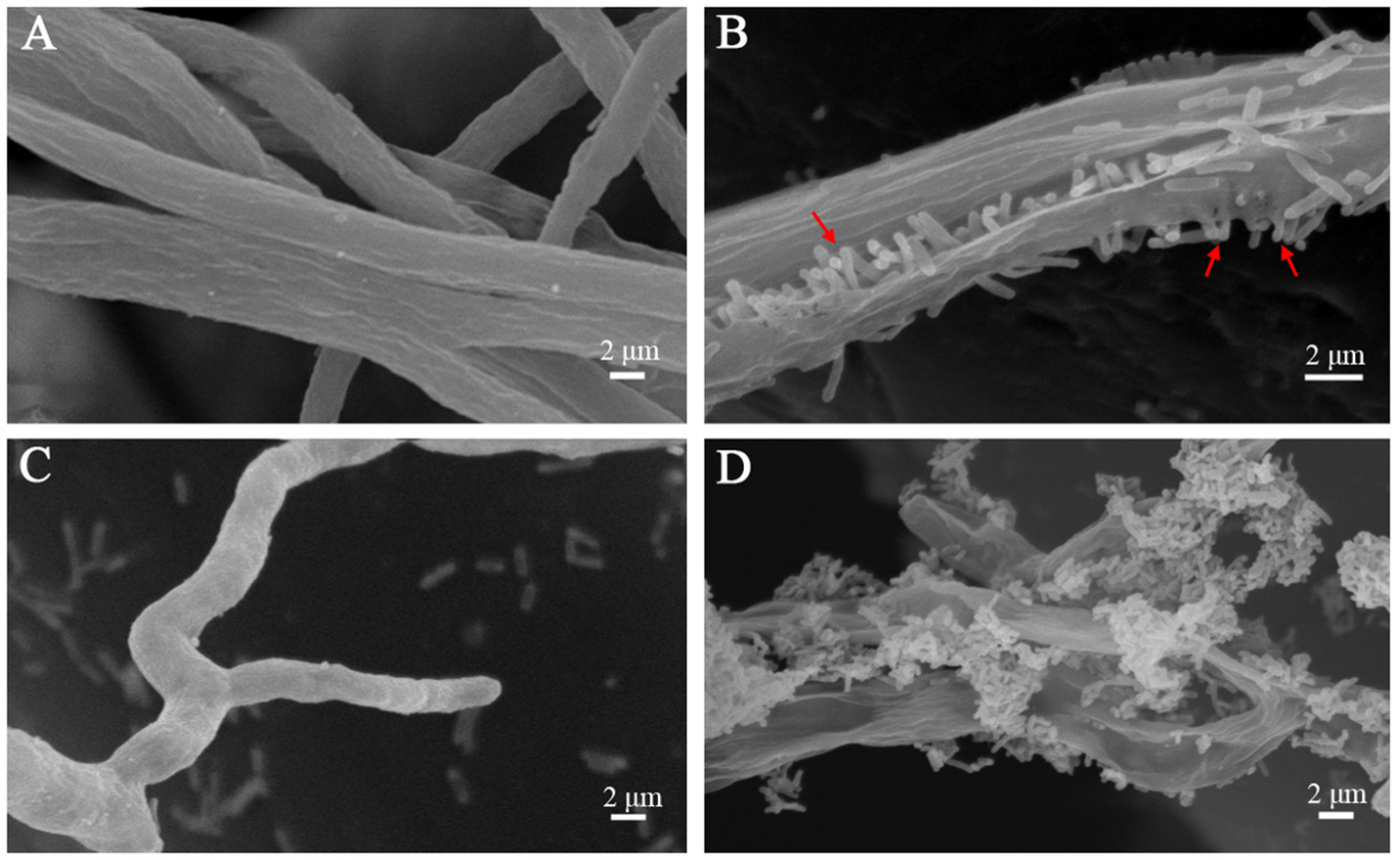
SEM examination of the *Pythium* hyphae and HSAF non-producer *Lysobacter* mutants during oomycete-bacterium physical interactions. **(A)** Control, *P. aphanidermatum* alone; **(B)** *L. enzymogenes* OH11 wild-type with *P. aphanidermatum* for 4 h, arrows show OH11 cells into the hyphae; **(C)** *L. enzymogenes* mutant K19 with *P. aphanidermatum* for 4 h; **(D)** *L. enzymogenes* mutant 5E4 with *P. aphanidermatum* for 4 h.

To achieve a better understanding of the interactions between *L. enzymogenes* and *P. aphanidermatum*, we developed a contact experiment. We immersed a small block of freshly grown hyphae of *P. aphanidermatum* into OH11 suspension for 10, 30 min, 2, 4, and 6 h, and then used SEM to examine the changes occurred to the oomycete and bacterium. SEM revealed a process of attachment, invasion and degradation of bacteria to the hyphae (Figure 5). During the first 10 min, the bacterial cells started to attach to the hyphae (Figure 5B); after 30 min, more bacterial cells aggregated on the mycelia (Figure 5C). The OH11 cells appeared to invade into the mycelia after 2 h (Figure 5D); between 4 and 6 h, more bacteria were observed in or on the oomycetes hyphae (Figure 5E), and finally the hyphae were degraded (Figure 5F). To investigate the role of HSAF in the interactions of *Lysobacter-Pythium*, we also conducted the experiments with HSAF-nonproducing *L. enzymogenes* strains K19 and 5E4 interacting with *P. aphanidermatum* for 4 h, using OH11 wild-type with *P. aphanidermatum* as a positive control (Figure 6B), *P. aphanidermatum* cultured alone served as a negative control (Figure 6A). Strain K19 could not adhere onto hyphae (Figure 6C), whereas strain 5E4 aggregated together but could not invade or lyse the mycelia (Figure 6D). The phenotype that strain 5E4 aggregated together was consistent with the previous study (Kobayashi et al., 2005). This result suggests that HSAF may be a key factor for the observed adhesion and invasion.

It has been revealed that *L. enzymogenes* SB-K88 perpendicularly attach to and densely colonize on the surface of *Aphanomyces cochlioides* hyphae (Islam et al., 2005), but it is unknown whether *Lysobacter* spp. can penetrate the hyphae of fungi using their lytic antibiotics or enzymes. Our study showed that the *L. enzymogenes* strain OH11 could attach, penetrate and lyse the hyphae of *P. aphanidermatum*. Additionally, we revealed that the antimycotic factor HSAF might play a crucial role in the interactions between *L. enzymogenes* and *P. aphanidermatum*. However, whether HSAF is solely as an antimycotic factor or also a signaling molecule is unclear. In future studies, we can further investigate the molecular mechanisms involved in contact interactions between *L. enzymogenes* and *P. aphanidermatum*.

## Conclusion

In this study, we investigated the potential effects of *P. aphanidermatum* on *L. enzymogenes*. Our data showed that the presence of *P. aphanidermatum* affected the expression of a wide range of genes spanning many functional groups and improved HSAF production and twitching motility of *Lysobacter*. Our data also demonstrated that *L. enzymogenes* detected and responded to the presence of oomycetes early (at 24 h), but the alteration of gene expression mainly occurred when bacteria were closer to the oomycetes (at 96 h). In summary, our results demonstrated that the biocontrol bacterium, *L. enzymogenes* OH11, showed transcriptional and antagonistic responses to a plant-pathogenic oomycete, *P. aphanidermatum*, and the antimycotic compound HSAF from *L. enzymogenes* may be involved in the responses. This work may provide new insights into the antagonistic strategies and genes involved in microbial interactions.

## Author contributions

YZ and YC carried out the experiments. GQ and FL designed and conducted the experiments. YZ, GQ, and FL contributed to the writing of the paper, LD revised the manuscript.

### Conflict of interest statement

The authors declare that the research was conducted in the absence of any commercial or financial relationships that could be construed as a potential conflict of interest.
