# Supplementary material for: Transcriptional and Antagonistic Responses of Biocontrol Strain Lysobacter enzymogenes OH11 to the Plant Pathogenic Oomycete Pythium aphanidermatum
Source: Front Microbiol. 2017 Jun 6;8:1025. doi: 10.3389/fmicb.2017.01025 (PMC5459918; doi:10.3389/fmicb.2017.01025)
Supplement: Supplementary file 1 [file DataSheet1.PDF]

Table S1 The differentially expressed genes of *L. enzymogenes* in the presence of *P. aphanidermatum* at 24 h, 48 h and 96 h, respectively (fold change≥2 or ≤0.5)

Class	Interaction time (numbers of gene)	Gene ID	Fold change	Function
I (Material transport and metabolism)	24h (13)	LysEGL000448	2.5671	General substrate transporter [Burkholderia sp. H160]
		LysEGL000573	2.6605	fimbrial subunit protein [Chromobacterium violaceum ATCC 12472]
		LysEGL000904	2.0746	hypothetical glycosidase protein [Xanthomonas albilineans]
		LysEGL001124	3.8741	putative glycosyltransferase protein [Xanthomonas albilineans]
		LysEGL002649	2.3545	Ox1 [Lysobacter enzymogenes]
		LysEGL002651	3.1719	hybrid polyketide synthase and nonribosomal peptide synthetase [Lysobacter enzymogenes]
		LysEGL003005	2.7149	probable transmembrane protein [Oceanicola granulosus HTCC2516]
		LysEGL003025	2.5844	Beta-N-acetylhexosaminidase [Flavobacterium johnsoniae UW101]
		LysEGL003267	2.17	beta-1,3-glucanase A [Lysobacter enzymogenes]
		LysEGL004434	2.6574	beta-1,3-glucanase [Lysobacter enzymogenes]
		LysEGL001318	0.4071	DSBA oxidoreductase [Acidovorax delafieldii 2AN]
		LysEGL005213	0.3983	ABC-type transport system, ATPase component [uncultured bacterium]
		LysEGL002652	0.4566	sterol desaturase-like protein [Lysobacter enzymogenes]
	48h (17)	LysEGL000877	2.383	glucokinase [Xanthomonas campestris pv. vasculorum NCPPB702]
		LysEGL000879	2.8658	putative n-acetyl-glucosamine transporter protein [Xanthomonas albilineans]
		LysEGL001192	2.1472	glucose-methanol-choline oxidoreductase [Shewanella baltica OS185]
		LysEGL004694	2.1263	Homoserine dehydrogenase [Stenotrophomonas maltophilia R551-3]
		LysEGL004917	2.1369	5-methyltetrahydropteroyltriglutamate--homocysteine methyltransferase [Xylella fastidiosa Temecula1]
		LysEGL005055	2.5976	threonine dehydratase [Xanthomonas oryzae pv. oryzae MAFF 311018]
		LysEGL005056	2.5174	2-isopropylmalate synthase [Xanthomonas campestris pv. musacearum NCPPB4381]
		LysEGL005057	3.1632	3-isopropylmalate dehydratase, large subunit [Stenotrophomonas maltophilia R551-3]

	LysEGL005058	3.305	probable 3-isopropylmalate dehydratase small subunit protein [Xanthomonas albilineans]
	LysEGL005060	3.4322	probable 3-isopropylmalate dehydrogenase protein [Xanthomonas albilineans]
	LysEGL003463	0.4839	MprA [uncultured bacterium pTW2]
	LysEGL003465	0.3175	MprA [uncultured bacterium pTW2]
	LysEGL003918	0.4195	imidazole glycerol-phosphate dehydratase/histidinol phosphatase [Xanthomonas axonopodis pv. citri str. 306]
	LysEGL003920	0.4333	histidinol dehydrogenase [Xanthomonas campestris pv. campestris str. ATCC 33913]
	LysEGL004590	0.4144	NADPH-sulfite reductase flavoprotein subunit [Xanthomonas campestris pv. vasculorum NCPPB702]
	LysEGL004595	0.4034	siroheme synthase [Bordetella petrii DSM 12804]
	LysEGL005220	0.3679	amino acid transporter LysE [Oxalobacter formigenes OXCC13]
96h (185)	LysEGL000328	2.2089	probable long-chain fatty acid outer membrane transporter, fadl protein [Xanthomonas albilineans]
	LysEGL000355	3.441	deoxyuridine 5'-triphosphate nucleotidohydrolase [Xanthomonas campestris pv. campestris str. ATCC 33913]
	LysEGL000356	4.0844	probable coenzyme a biosynthesis bifunctional protein coabc (dna/pantothenate metabolism flavoprotein) [Xanthomonas albilineans]
	LysEGL000357	3.3592	bifunctional phosphopantothenoylcysteine decarboxylase/phosphopantothenate synthase [Xanthomonas campestris pv. campestris str. B100]
	LysEGL000362	4.1971	Gfo/Idh/MocA family oxidoreductase [Methylococcus capsulatus str. Bath]
	LysEGL000425	2.8561	AMP-ligase [Xanthomonas axonopodis pv. citri str. 306]
	LysEGL000471	10.3171	glutamine amidotransferase [Xylella fastidiosa 9a5c]
	LysEGL000479	2.2398	ferrochelatase [Xanthomonas campestris pv. campestris str. B100]

	LysEGL000518	2.0878	drug:H+ antiporter-1 family protein [Xanthomonas campestris pv. vasculorum NCPPB702]
	LysEGL000519	2.0796	glutamate synthase subunit alpha [Xanthomonas campestris pv. campestris str. ATCC 33913]
	LysEGL000529	2.3695	8-amino-7-oxononanoate synthase [Xylella fastidiosa Dixon]
	LysEGL000536	2.0524	4-hydroxybenzoate octaprenyltransferase [Xanthomonas campestris pv. campestris str. ATCC 33913]
	LysEGL000810	2.0749	pyridoxal-5'-phosphate-dependent protein beta subunit [Methylobacterium radiotolerans JCM 2831]
	LysEGL000981	2.1361	copper/zinc superoxide dismutase [Stenotrophomonas sp. SKA14]
	LysEGL000993	7.7692	putative uroporphyrinogen-III synthase protein [Xanthomonas albilineans]
	LysEGL001042	4.2025	phosphate-selective porin O and P [Methylovorus sp. SIP3-4]
	LysEGL001045	2.5321	ABC-type oligopeptide transporter, permease protein [Aeromonas salmonicida subsp. salmonicida A449]
	LysEGL001094	2.4461	glutathione-regulated potassium-efflux protein C [Xanthomonas campestris pv. musacearum NCPPB4381]
	LysEGL001153	3.3714	transaldolase [Alteromonas macleodii 'Deep ecotype']
	LysEGL001214	2.3389	putative AMP nucleosidase [Stenotrophomonas maltophilia K279a]
	LysEGL001222	3.8228	hypothetical polysaccharide deacetylase protein [Xanthomonas albilineans]
	LysEGL001314	2.5503	major facilitator superfamily MFS_1 [Stenotrophomonas maltophilia R551-3]
	LysEGL001318	3.8467	DSBA oxidoreductase [Acidovorax delafieldii 2AN]
	LysEGL001394	6.612	probable proline racemase protein [Xanthomonas albilineans]
	LysEGL001447	4.0001	porphobilinogen deaminase [Xanthomonas axonopodis pv. citri str. 306]
	LysEGL001466	2.3134	diaminopimelate epimerase [Xanthomonas campestris pv. campestris str. ATCC 33913]
	LysEGL001549	4.6183	Leucyl aminopeptidase [Stenotrophomonas maltophilia R551-3]
	LysEGL001609	4.3644	putative phospholipid N-methyltransferase [Rhizobium leguminosarum bv. viciae 3841]
	LysEGL001632	2.2217	putative transmembrane sodium/calcium exchanger protein [Stenotrophomonas maltophilia

		K279a]
LysEGL001633	2.7142	Na+/H antiporter NhaA [Aeromonas salmonicida subsp. salmonicida A449]
LysEGL001720	2.3888	phosphatidylserine synthase [Xanthomonas campestris pv. campestris str. ATCC 33913]
LysEGL001798	2.2454	dephospho-CoA kinase [Stenotrophomonas maltophilia R551-3]
LysEGL001925	4.3362	adenosylmethionine--8-amino-7-oxononanoate transaminase [Xanthomonas campestris pv. campestris str. ATCC 33913]
LysEGL001966	3.6184	2-octaprenyl-6-methoxyphenyl hydroxylase [Xanthomonas campestris pv. campestris str. B100]
LysEGL001967	4.137	2-octaprenyl-3-methyl-6-methoxy-1,4-benzoquinol hydroxylase [Xanthomonas campestris pv. vasculorum NCPPB702]
LysEGL001979	2.6371	adenine phosphoribosyltransferase [Xanthomonas campestris pv. campestris str. B100]
LysEGL002063	2.1587	HemA [uncultured bacterium pTW2]
LysEGL002160	2.1209	class II aldolase/adducin domain protein [Pseudomonas putida GB-1]
LysEGL002246	3.4959	phosphoribosylaminoimidazole carboxylase [Saccharophagus degradans 2-40]
LysEGL002248	3.9072	phosphoribosylaminoimidazole carboxylase ATPase subunit [Xanthomonas campestris pv. Musacearum NCPPB4381]
LysEGL002253	12.5408	short chain dehydrogenase [Xanthomonas campestris pv. campestris str. ATCC 33913]
LysEGL002473	3.6629	Polyprenyl synthetase [Stenotrophomonas maltophilia R551-3]
LysEGL002516	3.6591	cation diffusion facilitator family transporter [Thiomicrospira crunogena XCL-2]
LysEGL002535	4.9038	probable asparagine synthase b protein [Xanthomonas albilineans]
LysEGL002573	2.8936	putative enoyl-CoA hydratase [Stenotrophomonas maltophilia K279a]
LysEGL002588	4.6278	bifunctional riboflavin kinase/FMN adenylyltransferase [Xanthomonas campestris pv. Vasculorum NCPPB702]
LysEGL002675	3.436	probable phosphomethylpyrimidine kinase (hmp-p kinase) protein [Xanthomonas albilineans]

LysEGL002689	2.6509	3-methyl-2-oxobutanoate hydroxymethyltransferase [Xanthomonas axonopodis pv. citri str. 306]
LysEGL002690	2.1955	3-methyl-2-oxobutanoate hydroxymethyltransferase [Xanthomonas axonopodis pv. citri str. 306]
LysEGL002718	2.6636	putative enoyl-CoA hydratase/isomerase [Stenotrophomonas maltophilia K279a]
LysEGL002897	2.964	FimV protein [Xanthomonas axonopodis pv. citri str. 306]
LysEGL002899	2.0181	N-(5'-phosphoribosyl)anthranilate isomerase [Xanthomonas campestris pv. campestris str. ATCC 33913]
LysEGL002987	3.2082	Dihydrodipicolinate reductase [Stenotrophomonas maltophilia R551-3]
LysEGL003038	2.2062	long-chain fatty acyl CoA ligase [Stenotrophomonas maltophilia]
LysEGL003098	3.5364	Acyl-CoA dehydrogenase [Idiomarina baltica OS145]
LysEGL003261	2.1533	glycosyltransferase 36 [Solibacter usitatus Ellin6076]
LysEGL003270	2.3027	putative AMP-binding protein [Burkholderia cenocepacia J2315]
LysEGL003271	2.9319	beta-glucosidase [Xanthomonas campestris pv. vesicatoria str. 85-10]
LysEGL003298	3.7514	Xylose isomerase domain protein TIM barrel [Stenotrophomonas maltophilia R551-3]
LysEGL003411	3.4715	putative methyltransferase [Xanthomonas campestris pv. campestris str. B100]
LysEGL003412	4.161	5-methyltetrahydrofolate-homocysteine methyl transferase [Xanthomonas oryzae pv. oryzae MAFF 311018]
LysEGL003489	2.5609	putative dihydrokaempferol 4-reductase (NAD-dependent epimerase/dehydratase) [Bradyrhizobium sp. ORS278]
LysEGL003630	5.6963	chorismate mutase [Pseudomonas aeruginosa PAO1]
LysEGL003631	4.5756	molybdopterin biosynthesis protein MoeB [Xanthomonas campestris pv. vesicatoria str. 85-10]
LysEGL003648	2.1843	FAD dependent oxidoreductase [Burkholderia sp. CCGE1001]
LysEGL003673	2.2194	pterin-4-alpha-carbinolamine dehydratase [Xanthomonas campestris pv. campestris str. B100]
LysEGL003701	2.8919	putative acyl carrier protein phosphodiesterase [Stenotrophomonas maltophilia K279a]
LysEGL003706	2.3023	3-phosphoshikimate 1-carboxyvinyltransferase [Xanthomonas campestris pv. campestris str. B100]
LysEGL003785	2.344	putative ThiF domain protein [Stenotrophomonas maltophilia K279a]

	LysEGL003864	2.5602	diadenosine tetraphosphate (Ap4A) hydrolase and other HIT family hydrolases [Magnetospirillum magneticum AMB-1]
	LysEGL003874	2.7156	hydroxymethylglutaryl-CoA lyase [Stenotrophomonas sp. SKA14]
	LysEGL003919	5.2616	probable histidinol-phosphate aminotransferase (ihpat) protein [Xanthomonas albilineans]
	LysEGL003920	14.0833	histidinol dehydrogenase [Xanthomonas campestris pv. campestris str. ATCC 33913]
	LysEGL003921	25.2864	probable atp phosphoribosyltransferase (atp-prt) protein [Xanthomonas albilineans]
	LysEGL004098	2.2144	hypoxanthine-guanine phosphoribosyltransferase [Xanthomonas axonopodis pv. citri str. 306]
	LysEGL004122	4.3663	probable thymidylate kinase (dtmp kinase) protein [Xanthomonas albilineans]
	LysEGL004123	2.8019	aminotransferase, class IV superfamily [Methylophaga thiooxidans DMS010]
	LysEGL004127	2.1335	acyl-carrier-protein S-malonyltransferase [Xanthomonas campestris pv. campestris str. ATCC 33913]
	LysEGL004169	3.0604	probable molybdenum cofactor biosynthesis protein a [Xanthomonas albilineans]
	LysEGL004246	2.1554	putative phosphoribosylglycinamide formyltransferase 2 [Stenotrophomo nas maltophilia K279a]
	LysEGL004375	2.9498	D-amino-acid dehydrogenase [Xanthomonas campestris pv. campestris str. B100]
	LysEGL004444	7.7067	Fatty-acid desaturase [Hahella chejuensis KCTC 2396]
	LysEGL004590	4.2925	NADPH-sulfite reductase flavoprotein subunit [Xanthomonas campestris pv. vasculorum NCPPB702]
	LysEGL004592	3.4819	sulfite reductase subunit beta [Xanthomonas oryzae pv. oryzae KACC10331]
	LysEGL004595	9.7748	siroheme synthase [Bordetella petrii DSM 12804]
	LysEGL004596	3.8813	cysteine synthase A [Stenotrophomonas maltophilia R551-3]
	LysEGL004638	5.5029	putative phosphotransferase system (pts), mannose/fructose-specific component IIa protein [Xanthomonas albilineans]
	LysEGL004639	2.8701	phosphotransferase system HPr enzyme [Xanthomonas axonopodis pv. citri str. 306]
	LysEGL004640	3.6159	phosphotransferase system enzyme I [Xanthomonas campestris pv. campestris str. ATCC 33913]
	LysEGL004658	15.1593	pyrroline-5-carboxylate reductase [Stenotrophomonas sp. SKA14]
	LysEGL004669	2.0524	shikimate kinase [Stenotrophomonas sp. SKA14]

	LysEGL004670	2.4415	3-dehydroquinate synthase [Xanthomonas campestris pv. campestris str. ATCC 33913]
	LysEGL004685	2.8664	L-serine dehydratase [Xanthomonas campestris pv. campestris str. ATCC 33913]
	LysEGL004694	30.2053	Homoserine dehydrogenase [Stenotrophomonas maltophilia R551-3]
	LysEGL004695	13.5523	cystathionine gamma-synthase [Xanthomonas oryzae pv. oryzae KACC10331]
	LysEGL004696	10.7111	homoserine O-acetyltransferase [Xanthomonas campestris pv. campestris str. ATCC 33913]
	LysEGL004722	2.6746	acyl-CoA dehydrogenase [Xanthomonas axonopodis pv. citri str. 306]
	LysEGL004726	4.6254	putative transport-related membrane protein [Pseudomonas fluorescens SBW25]
	LysEGL004809	2.3911	putative fumarylacetoacetate hydrolase family protein [Xanthomonas albilineans]
	LysEGL004821	4.6803	cysteine synthase [Xanthomonas campestris pv. vasculorum NCPPB702]
	LysEGL004975	2.6466	hypothetical protein XALc_0794 [Xanthomonas albilineans]
	LysEGL005005	9.7364	thiamine-phosphate pyrophosphorylase [Xylella fastidiosa 9a5c]
	LysEGL005006	2.0952	glutamate-1-semialdehyde aminotransferase [Xanthomonas campestris pv. vasculorum NCPPB702]
	LysEGL005009	9.4261	bifunctional N-succinyldiaminopimelate-aminotransferase/acetylornithine transaminase protein [Xanthomonas axonopodis pv. citri str. 306]
	LysEGL005020	2.0259	phosphofructokinase [Stenotrophomonas maltophilia R551-3]
	LysEGL005045	3.8563	bifunctional aspartokinase I/homeserine dehydrogenase I [Xanthomonas campestris pv. vasculorum NCPPB702]
	LysEGL005047	3.4261	homoserine kinase [Xanthomonas campestris pv. vasculorum NCPPB702]
	LysEGL005051	3.621	ketol-acid reductoisomerase [Xanthomonas campestris pv. campestris str. ATCC 33913]
	LysEGL005052	5.1162	acetolactate synthase, large subunit, biosynthetic type [Stenotrophomonas sp. SKA14]
	LysEGL005054	3.7568	branched-chain amino acid aminotransferase [Meiothermus ruber DSM 1279]
	LysEGL005055	5.0192	threonine dehydratase [Xanthomonas oryzae pv. oryzae MAFF 311018]
	LysEGL005056	2.2174	2-isopropylmalate synthase [Xanthomonas campestris pv. musacearum NCPPB4381]
	LysEGL005057	3.4588	3-isopropylmalate dehydratase, large subunit [Stenotrophomonas maltophilia R551-3]

LysEGL005058	2.3477	probable 3-isopropylmalate dehydratase small subunit protein [Xanthomonas albilineans]
LysEGL005060	3.2653	probable 3-isopropylmalate dehydrogenase protein [Xanthomonas albilineans]
LysEGL005139	2.259	putative FecR protein [Stenotrophomonas sp. SKA14]
LysEGL005214	2.4221	Acyl-CoA thioesterase I / Lysophospholipase [Ralstonia solanacearum UW551]
LysEGL000039	0.4398	acyl-CoA dehydrogenase [Xanthomonas campestris pv. vasculorum NCPPB702]
LysEGL000118	0.2783	beta-1,3-glucanase C [Lysobacter enzymogenes]
LysEGL000212	0.3225	chitinase A [Xanthomonas sp. AK]
LysEGL000234	0.3535	N-acylglucosamine 2-epimerase [Stenotrophomonas sp. SKA14]
LysEGL000448	0.2427	General substrate transporter [Burkholderia sp. H160]
LysEGL000615	0.4552	putative glutathionylspermidine synthase [Xanthomonas campestris pv. musacearum NCPPB4381]
LysEGL000619	0.4423	potassium channel related protein [Xanthomonas campestris pv. musacearum NCPPB4381]
LysEGL000820	0.4782	probable glucans biosynthesis protein d precursor [Xanthomonas albilineans]
LysEGL000853	0.4942	phospholipase [Stenotrophomonas sp. SKA14]
LysEGL000900	0.3607	putative TonB dependent receptor [Stenotrophomonas maltophilia K279a]
LysEGL000970	0.4349	TonB-dependent receptor [Stenotrophomonas maltophilia R551-3]
LysEGL001056	0.4251	xanthomonadin biosynthesis protein [Xanthomonas campestris pv. vesicatoria str. 85-10]
LysEGL001087	0.4073	Coproporphyrinogen oxidase [Stenotrophomonas maltophilia R551-3]
LysEGL001312	0.2808	Alcohol acetyltransferase [Chitinophaga pinensis DSM 2588]
LysEGL001418	0.254	TonB-dependent siderophore receptor [Hirschia baltica ATCC 49814]
LysEGL001438	0.4582	putative cation efflux protein [Stenotrophomonas maltophilia K279a]
LysEGL001574	0.2798	coproporphyrinogen III oxidase, putative [Stigmatella aurantiaca DW4/3-1]
LysEGL000904	0.4041	hypothetical glycosidase protein [Xanthomonas albilineans]
LysEGL001192	0.3341	glucose-methanol-choline oxidoreductase [Shewanella baltica OS185]
LysEGL001576	0.2448	carbonyl reductase [NADPH] 1 (nadph-dependent carbonylreductase 1) (prostaglandin-e(2) 9-reductase) [Stigmatella aurantiaca DW4/3-1]

	LysEGL001624	0.4893	putative peptide transport protein [Stenotrophomonas maltophilia K279a]
	LysEGL001713	0.4449	putative ATP-binding component of ABC transporter [Stenotrophomonas maltophilia K279a]
	LysEGL002038	0.2139	xanthomonalisin [Stigmatella aurantiaca DW4/3-1]
	LysEGL002177	0.3234	TonB-dependent receptor [Xanthomonas axonopodis pv. citri str. 306]
	LysEGL002180	0.2587	cellulase, putative, cel5G [Cellvibrio japonicus Ueda107]
	LysEGL002191	0.432	putative TonB dependent receptor [Stenotrophomonas maltophilia K279a]
	LysEGL002206	0.4715	putative dipeptidyl peptidase [Xanthomonas campestris pv. vesicatoria str. 85-10]
	LysEGL002338	0.4025	protein-export membrane protein SecF [Stenotrophomonas maltophilia R551-3]
	LysEGL002556	0.3591	ABC transporter phosphate permease [Xanthomonas campestris pv. campestris str. ATCC 33913]
	LysEGL002607	0.4313	formyl transferase domain-containing protein [Acaryochloris marina MBIC11017]
	LysEGL002646	0.1717	Ox4 [Lysobacter enzymogenes]
	LysEGL002647	0.2135	Ox3 [Lysobacter enzymogenes]
	LysEGL002648	0.1904	Ox2 [Lysobacter enzymogenes]
	LysEGL002649	0.1852	Ox1 [Lysobacter enzymogenes]
	LysEGL002651	0.2333	hybrid polyketide synthase and nonribosomal peptide synthetase [Lysobacter enzymogenes]
	LysEGL002652	0.3319	sterol desaturase-like protein [Lysobacter enzymogenes]
	LysEGL002655	0.4136	TonB-dependent outer membrane receptor precursor [Xanthomonas campestris pv. campestris str. B100]
	LysEGL002883	0.4502	copper homeostasis protein (lipoprotein) [Photorhabdus asymbiotica]
	LysEGL003005	0.1276	probable transmembrane protein [Oceanicola granulosus HTCC2516]
	LysEGL003023	0.4411	TonB-dependent receptor domain protein [Stenotrophomonas sp. SKA14]
	LysEGL003024	0.1503	hypothetical protein XAC4355 [Xanthomonas axonopodis pv. citri str. 306]
	LysEGL003025	0.1912	Beta-N-acetylhexosaminidase [Flavobacterium johnsoniae UW101]
	LysEGL003028	0.3511	beta-mannosidase [Stenotrophomonas sp. SKA14]
	LysEGL003091	0.4016	Ornithine carbamoyltransferase [Thioalkalivibrio sp. HL-EbGR7]
	LysEGL003263	0.3864	glycosyltransferase 36 [Solibacter usitatus Ellin6076]

	LysEGL003267	0.192	beta-1,3-glucanase A [Lysobacter enzymogenes]
	LysEGL003277	0.4019	ABC transporter related [Clostridium cellulolyticum H10]
	LysEGL003281	0.4171	TonB-dependent receptor [Cellvibrio japonicus Ueda107]
	LysEGL003282	0.4157	major facilitator superfamily MFS_1 [Pedobacter heparinus DSM 2366]
	LysEGL003463	0.1612	MprA [uncultured bacterium pTW2]
	LysEGL003465	0.2088	MprA [uncultured bacterium pTW2]
	LysEGL003500	0.2789	ABC transporter sugar permease [Xanthomonas campestris pv. vesicatoria str. 85-10]
	LysEGL003501	0.3267	ABC transporter sugar permease [Xylella fastidiosa 9a5c]
	LysEGL003502	0.3194	probable abc transporter sugar-binding protein [Xanthomonas albilineans]
	LysEGL003727	0.4594	arsenate reductase [Pseudomonas syringae pv. oryzae str. 1_6]
	LysEGL003902	0.4739	Amino acid permease-associated region [Xylella fastidiosa Ann-1]
	LysEGL003903	0.3373	putative amino acid-polyamine-organocation (apc) superfamily transporter protein [Xanthomonas albilineans]
	LysEGL003944	0.4439	alpha-1,2-mannosidase [Streptomyces sp. AA4]
	LysEGL003971	0.2786	FAD-dependent pyridine nucleotide-disulphide oxidoreductase [Geodermatophilus obscurus DSM 43160]
	LysEGL003973	0.4081	amino acid adenylation domain-containing protein [Nostoc punctiforme PCC 73102]
	LysEGL003984	0.3955	argininosuccinate synthase [Xanthomonas campestris pv. ca mpestris str. ATCC 33913]
	LysEGL004088	0.1246	alpha-lytic protease [Lysobacter enzymogenes]
	LysEGL004434	0.4161	beta-1,3-glucanase [Lysobacter enzymo genes]
	LysEGL004576	0.1909	TonB-dependent receptor [Xanthomonas axonopodis pv. citri str. 306]
	LysEGL004758	0.3525	outer membrane receptor protein, mostly Fe transport [Stenotrophomonas sp. SKA14]
	LysEGL004962	0.3858	TonB dependent receptor, putative [Rhodospirillum centenum SW]
	LysEGL005106	0.3937	probable acetyl-coa carboxylase, biotin carboxylase subunit protein [Xanthomonas albilineans]

		LysEGL005156	0.4843	putative vitamin B12 receptor protein [Steno trophomonas maltophilia K279a]
II (transcription)	48h (5)	LysEGL001359	2.2838	RNA polymerase, sigma 28 subunit, FliA/WhiG family [Burkholderia ambifaria MEX-5]
		LysEGL001044	0.3451	Putative transcriptional regulator YrzC [Methylacidiphilum infernorum V4]
		LysEGL002957	0.4984	transcriptional regulators-like protein [Burkholderia ambifaria AMMD]
		LysEGL002981	0.4315	probable heat-inducible transcriptional repressor protein [Xanthomonas albilineans]
		LysEGL004443	0.4489	transcription elongation factor, putative [Methylococcus capsulatus str. Bath]
	96h (33)	LysEGL000014	2.7306	transcriptional regulator, putative [Oceanicaulis alexandrii HTCC2633]
		LysEGL000055	4.2696	AraC family transcriptional regulator [Solibacter usitatus Ellin6076]
		LysEGL000084	3.139	probable transcriptional regulator protein, TetR family [Rhizobium etli CIAT 652]
		LysEGL000327	50.3441	transcriptional regulator, MarR family [Meiothermus silvanus DSM 9946]
		LysEGL000394	2.0558	GntR family transcriptional regulator [Burkholderia xenovorans LB400]
		LysEGL000665	2.5864	putative LysR family regulatory protein [Stenotrophomonas maltophilia K279a]
		LysEGL000827	2.4909	ECF subfamily RNA polymerase sigma-24 factor [Burkholderia cenocepacia MC0-3]
		LysEGL001044	8.8072	Putative transcriptional regulator YrzC [Methylacidiphilum infernorum V4]
		LysEGL001127	4.509	transcriptional regulator [Mesorhizobium loti MAFF303099]
		LysEGL001193	3.9309	DeoR family transcriptional regulator [Streptomyces hygroscopicus ATCC 53653]
		LysEGL001627	5.708	probable transcriptional regulator, marr family protein [Xanthomonas albilineans]
		LysEGL001669	2.8902	acetyltransferase, gnat family [Pseudovibrio sp. JE062]
		LysEGL002007	2.8751	RNA polymerase factor sigma-70 [Xanthomonas campestris pv. vasculorum NCPPB702]
		LysEGL002014	3.6181	transcriptional regulator [Xanthomonas campestris pv. campestris str. ATCC 33913]
		LysEGL002230	2.7639	transcriptional regulator, TetR family [bacterium S5]
		LysEGL002517	2.2301	methicillin resistance protein [Xanthomonas axonopodis pv. citri str. 306]
		LysEGL002518	7.8435	BlaR1 peptidase M56 family protein [Xanthomonas campestris pv. vesicatoria str. 85-10]
		LysEGL002833	10.8355	putative MerR family trasncriptional regulator SoxR protein [Stenotrophomonas maltophilia K279a]
		LysEGL002981	2.2161	probable heat-inducible transcriptional repressor protein [Xanthomonas albilineans]

		LysEGL003053	3.0866	putative SpoVT/AbrB domain transcriptional regulatory protein [Stenotrophomon as maltophilia K279a]
		LysEGL003441	6.0134	acetyltransferase, GNAT family [Stenotrophomonas sp. SKA14]
		LysEGL003481	2.3597	transcriptional repressor, CopY family [Stenotrophomonas maltophilia R551-3]
		LysEGL003520	4.5706	TetR family transcriptional regulator [Serratia proteamaculans 568]
		LysEGL003689	2.9443	transcriptional regulator, HxlR family [Dickeya zeae Ech1591]
		LysEGL004170	2.7517	probable 3-deoxy-d-manno-octulosonic acid kinase (kdo kinase) protein [Xanthomonas albilineans]
		LysEGL004393	3.2783	ArsR family transcriptional regulator [Janthinobacterium sp. Marseille]
		LysEGL004417	2.6493	transcriptional regulator of molybdate metabolism, LysR family [Lutiella nitroferrum 2002]
		LysEGL004461	6.717	LysR family transcriptional regulator [Myxococcus xanthus DK 1622]
		LysEGL004594	4.5097	transcriptional regulator, LysR family [Stenotrophomonas maltophilia R551-3]
		LysEGL004854	2.6401	sigma-54 interacting transcription regulator protein [Ralstonia solanacearum GMI1000]
		LysEGL004884	2.2352	metal dependent phosphohydrolase [Burkholderia phymatum STM815]
		LysEGL004965	2.185	transcriptional regulator, AraC family [Burkholderia ambifaria MEX-5]
		LysEGL005140	2.1846	putative RNA polymerase sigma factor [Stenotrophomonas maltophilia K279a]
III	24h (1)	LysEGL005226	0.4774	universal stress protein family [Brevundimonas sp. BAL3]
(signal transduction)	48h (3)	LysEGL003923	2.249	response regulator receiver domain protein (CheY-like) [Marinobacter algicola DG893]
		LysEGL002947	0.4458	periplasmic sensor signal transduction histidine kinase [Alkalilimnicola ehrlichii MLHE-1]
		LysEGL005226	0.4931	universal stress protein family [Brevundimonas sp. BAL3]
	96h (25)	LysEGL000422	7.7586	phage shock protein A, PspA [Pseudomonas mendocina ymp]
		LysEGL000663	2.1485	bifunctional isocitrate dehydrogenase kinase/phosphatase protein [Xanthomonas campestris pv. musacearum NCPPB4381]
		LysEGL000745	2.0689	sigma54 specific transcriptional regulator, Fis family protein [Burkholderia ubonensis Bu]
		LysEGL000918	2.0153	sensor kinase [Lysobacter enzymogenes]
		LysEGL000969	4.8665	two-component system sensor protein [Xanthomonas axonopodis pv. citri str. 306]

		LysEGL000978	3.7648	putative nitrogen regulation protein nr(ii) [Steno trophomonas maltophilia K279a]
		LysEGL000979	3.1702	probable two-component regulatory system regulatory ntrc transcription regulator protein [Xanthomonas albilineans]
		LysEGL001422	2.9977	periplasmic sensor signal transduction histidine kinase [Burkholderia xenovorans LB400]
		LysEGL001446	4.9435	probable two-component system regulatory protein [Xanthomonas albilineans]
		LysEGL001607	5.2162	GAF domain/GGDEF domain protein [Acidobacterium capsulatum ATCC 51196]
		LysEGL001610	2.848	two-component response regulator [Bradyrhizobium japonicum USDA 110]
		LysEGL002425	2.1535	serine/threonine protein kinase PpkA [Cellvibrio japonicus Ueda107]
		LysEGL002433	2.3908	putative sensor protein [Xanthomonas albilineans]
		LysEGL003201	2.0993	CheA signal transduction histidine kinase [Chthoniobacter flavus Ellin428]
		LysEGL003202	4.163	CheW purine-binding chemotaxis protein [Verrucomicrobium spinosum DSM 4136]
		LysEGL003204	2.1624	MCP methyltransferase, CheR-type [Pseudomonas fluorescens Pf0-1]
		LysEGL003590	2.137	two component transcriptional regulator, LuxR family [Stenotrophomonas maltophilia R551-3]
		LysEGL003816	2.3175	two component transcriptional regulator [Methylobacillus flagellatus KT]
		LysEGL004115	3.4309	putative regulatory protein [Xanthomonas albilineans]
		LysEGL000808	0.34	diguanylate cyclase/phosphodiesterase withMHYT sensor [Pantoea sp. At-9b]
		LysEGL000906	0.3842	two component system response regulator [Bordetella petrii DSM 12804]
		LysEGL003609	0.469	methyl-accepting chemotaxis protein [Xanthomonas campestris pv. vesicatoria str. 85-10]
		LysEGL004844	0.4125	two-component system sensor protein [Xanthomonas campestris pv. campestris str. ATCC 33913]
		LysEGL004986	0.2932	diguanylate cyclase/phosphodiesterase with PAS/PAC sensor(s) [Shewanella frigidimarina NCIMB 400]
		LysEGL005226	0.3729	universal stress protein family [Brevundimonas sp. BAL3]
IV (General function predicted only)	24h (6)	LysEGL000447	2.0648	putative secreted protein [Xanthomonas oryzae pv. oryzicola BLS256]
		LysEGL001258	2.6377	lysyl endopeptidase [Herpetosiphon aurantiacus ATCC 23779]
		LysEGL002035	2.341	lysyl endopeptidase precursor[Lysobacter sp. IB-9374]
		LysEGL001306	2.3343	Peptidase M23 [Micromonospora aurantiaca ATCC 27029]

	LysEGL003004	2.418	Heparinase II/III family protein [Desulfatibacillum alkenivorans AK-01]
	LysEGL004365	0.3946	Globin [Thioalkalivibrio sp. K90mix]
48h (11)	LysEGL000447	2.0113	putative secreted protein [Xanthomonas oryzae pv. oryzicola BLS256]
	LysEGL001049	4.7957	probable pteridine-dependent deoxygenase like protein [Xanthomonas albilineans]
	LysEGL001346	2.1517	R body protein RebB-like protein [Burkholderia sp. CCGE1003]
	LysEGL001347	2.2947	R body protein RebB-like protein [Burkholderia sp. CCGE1003]
	LysEGL002784	2.2209	lipase family protein [Cellvibrio japonicus Ueda107]
	LysEGL002839	2.233	Acetyltransferase (GNAT) family protein [Xanthomonas campestris pv. campestris str. B100]
	LysEGL003717	2.0844	putative_membrane_protein_pd-(d/e) xk_nuclease_superfamily [Xanthomonas albilineans]
	LysEGL001018	0.4329	transglycosylase-associated protein [Sphingopyxis alaskensis RB2256]
	LysEGL004727	0.4755	TrpR binding protein WrbA [Rhodopseudomonas palustris BisA53]
	LysEGL004586	0.4663	transglycosylase-associated protein [Psychrobacter cryohalolentis K5]
	LysEGL002958	0.4292	putative thiol-disulphide oxidoreductase DCC [Ralstonia metallidurans CH34
96h (97)	LysEGL000240	2.1659	putative membrane protein [Xanthomonas campestris pv. vasculorum NCPPB702]
	LysEGL000298	2.7154	putative ribonuclease [Stenotrophomonas maltophilia K279a]
	LysEGL000528	2.313	biotin biosynthesis protein BioH [Xanthomonas campestris pv. vesicatoria str. 85-10]
	LysEGL000672	2.7911	transporter of the DMT superfamily [Janthinobacterium sp. Marseille]
	LysEGL000868	3.3153	putative phospholipase accessory protein [Xanthomonas campestris pv. campestris str. B100]
	LysEGL000885	4.8643	short-chain dehydrogenase/reductase SDR [Chthoniobacter flavus Ellin428]
	LysEGL000989	2.8068	putative secreted esterase/lipase/thioesterase family protein [Xanthomonas campestris pv. vesicatoria str. 85-10]
	LysEGL001023	2.7232	transferase [Xanthomonas campestris pv. campestris str. ATCC 33913]
	LysEGL001092	3.8704	putative ankyrin-like membrane protein [Xanthomonas campestris pv. vesicatoria str. 85-10]
	LysEGL001157	5.1214	putative transmembrane protein [Stenotrophomonas maltophilia K279a]
	LysEGL001225	2.1642	lipolytic enzyme, G-D-S-L family [Stenotrophomonas sp. SKA14]

	LysEGL001464	3.1725	membrane protein [Stenotrophomonas sp. SKA14]
	LysEGL001444	2.4439	carboxylesterase [Xanthomonas campestris pv. musacearum NCPPB4381]
	LysEGL001478	2.3435	ElaA protein [Xanthomonas campestris pv. campestris str. ATCC 33913]
	LysEGL001638	2.2704	putative transmembrane protein [Stenotrophomonas maltophilia K279a]
	LysEGL001550	4.1384	hydrolase [Stenotrophomonas sp. SKA14]
	LysEGL001606	2.2907	Metallo-beta-lactamase superfamily protein [Xanthomonas campestris pv. campestris str. B100]
	LysEGL001674	4.5817	outer membrane protein [Xanthomonas campestris pv. campestris str. ATCC 33913]
	LysEGL001722	2.0656	ribosomal-protein-alanine acetyltransferase [Xanthomonas axonopodis pv. citri str. 306]
	LysEGL001887	2.2283	putative phenazine biosynthesis-like protein [Stenotrophomonas maltophilia K279a]
	LysEGL001899	2.1902	rhamnosyltransferase [Nitrosococcus oceani ATCC 19707]
	LysEGL001978	3.2143	Integral membrane protein DUF6 [Denitrovibrio acetiphilus DSM 12809]
	LysEGL002004	2.0314	putative secreted protein [Stenotrophomonas maltophilia R551-3]
	LysEGL002006	5.2215	putative transmembrane protein [Stenotrophomonas maltophilia K279a]
	LysEGL002045	2.2276	NAD(P)H: quinone oxidoreductase, type IV [Stenotrophomonas sp. SKA14]
	LysEGL002419	2.0466	LysM domain protein [Stenotrophomonas sp. SKA14
	LysEGL002467	3.4274	PmbA protein [Xanthomonas campestris pv. campestris str. ATCC 33913]
	LysEGL002612	2.4695	putative cytochrome c assembly transmembrane protein [Xanthomonas albilineans]
	LysEGL002779	5.2959	YCII domain-containing protein [Hyphomonas neptunium ATCC 15444]
	LysEGL002834	6.9165	alcohol dehydrogenase GroES domain protein [Streptosporangium roseum DSM 43021]
	LysEGL002992	2.2191	regulatory protein [Stenotrophomonas maltophilia R551-3]
	LysEGL003059	2.7034	putative nudix hydrolase family protein [Xanthomonas albilineans]
	LysEGL003099	2.5672	short chain dehydrogenase [Myxococcus xanthus DK 1622]
	LysEGL003108	4.3877	Csw015 [uncultured bacterium]
	LysEGL003174	3.8423	beta-lactamase domain-containing protein [Caulobacter sp. K31]
	LysEGL003296	2.3234	oxidoreductase domain protein [Stenotrophomonas maltophilia R551-3]
	LysEGL003297	2.2482	putative nucleoside H+ symport transporter permease protein [Stenotrophomonas maltophilia

		K279a]
LysEGL003456	5.0051	NAD [Pseudomonas aeruginosa PA7]
LysEGL003482	7.9824	peptidase M56 BlaR1 [Stenotrophomonas maltophilia R551-3]
LysEGL003588	2.6678	putative D--3-hydroxybutyrate oligomer hydrolase lipoprotein transmembrane [Marinobacter aquaeolei VT8]
LysEGL003664	8.3705	hydrolase [Pseudomonas syringae pv. syringae B728a]
LysEGL003710	2.2554	putative sulfite oxidase subunit YedY [Xanthomonas oryzae pv. oryzae MAFF 311018]
LysEGL003734	2.6928	pteridine reductase [Oceanospirillum sp. MED92]
LysEGL004003	4.894	putative GTP cyclohydrolase [Pseudomonas fluorescens Pf0-1]
LysEGL004005	2.8612	phosphoglycolate phosphatase [Xanthomonas axonopodis pv. citri str. 306]
LysEGL004208	2.1582	nitrilase [Xanthomonas campestris pv. campestris str. 8004]
LysEGL004226	8.6249	pirin-related protein [Xanthomonas campestris pv. campestris str. ATCC 33913]
LysEGL004229	4.9282	pirin-related protein [Xanthomonas campestris pv. vesicatoria str. 85-10]
LysEGL004240	2.3184	ribonuclease PH [Plesiocystis pacifica SIR-1]
LysEGL004247	2.4703	putative integral membrane protein [Shewanella frigidimarina NCIMB 400]
LysEGL004363	2.1854	periplasmic protein-like protein [Variovorax paradoxus S110]
LysEGL004462	2.2432	ATPase [Hahella chejuensis KCTC 2396]
LysEGL004490	2.1877	UDP-2,3-diacylglucosamine hydrolase [Stenotrophomonas sp. SKA14]
LysEGL004520	5.8477	Extradiol ring-cleavage dioxygenase class III protein subunit B [Tolumonas auensis DSM 9187]
LysEGL004782	3.472	GCN5-related N-acetyltransferase [Chitinophaga pinensis DSM 2588]
LysEGL005053	5.112	acetolactate synthase isozyme II small subunit [Xanthomonas campestris pv. campestris str. ATCC 33913]
LysEGL005189	2.6324	membrane protein-like protein [Psychromonas ingrahamii 37]
LysEGL004841	6.5862	degV family protein [Desulfovibrio salexigens DSM 2638]
LysEGL000074	0.4851	trans-2-enoyl-CoA reductase [Xanthomonas campestris pv. campestris str. ATCC 33913]
LysEGL000113	0.3569	heme utilization/adhesion protein [Burkholderia sp. CCGE1002]

LysEGL000447	0.2671	putative secreted protein [Xanthomonas oryzae pv. oryzicola BLS256]
LysEGL000671	0.4279	putative lipoprotein [Myxococcus xanthus DK 1622]
LysEGL001170	0.2894	chitin-binding domain 3 protein [Stenotrophomonas maltophilia R551-3]
LysEGL001306	0.2139	Peptidase M23 [Micromonospora aurantiaca ATCC 27029]
LysEGL000901	0.3432	sodium/glucose cotransport protein [Xanthomonas oryzae pv. oryzae MAFF 311018]
LysEGL000908	0.2245	electron transport protein SCO1/SenC [Delftia acidovorans SPH-1]
LysEGL001123	0.3518	Patatin [Rhodopseudomonas palustris DX-1]
LysEGL001258	0.2024	lysyl endopeptidase [Herpetosiphon aurantiacus ATCC 23779]
LysEGL001311	0.3659	rhamnosyltransferase [Burkholderia ambifaria MEX-5]
LysEGL001342	0.219	RebB like protein [Chromobacterium violaceum ATCC 12472]
LysEGL001346	0.2081	R body protein RebB-like protein [Burkholderia sp. CCGE1003]
LysEGL001347	0.1672	R body protein RebB-like protein [Burkholderia sp. CCGE1003]
LysEGL001577	0.1444	putative sulfotransferase protein [Stigmatella aurantiaca DW4/3-1]
LysEGL001597	0.4124	aminopeptidase [Stenotrophomonas sp. SKA14]
LysEGL001710	0.4924	putative secreted protein [Xanthomonas campestris pv. vesicatoria str. 85-10]
LysEGL001917	0.2689	putative transmembrane protein [Stenotrophomonas maltophilia K279a]
LysEGL002037	0.1955	lysyl endopeptidase precursor [Lysobacter sp. IB-9374]
LysEGL002040	0.2484	bacterial leucyl aminopeptidase [Stigmatella aurantiaca DW4/3-1]
LysEGL002119	0.4032	pyrroloquinoline quinone biosynthesis protein PqqE [Xanthomonas campestris pv. campestris str. ATCC 33913]
LysEGL002171	0.1705	Taurine catabolism dioxygenase TauD/TfdA [Burkholderia ubonensis Bu]
LysEGL002178	0.2562	SapC-related protein [Xanthomonas campestris pv. vesicatoria str. 85-10]
LysEGL002179	0.1625	Pass1-related protein [Xanthomonas oryzae pv. oryzae KACC10331]
LysEGL002542	0.1867	endoproteinase Arg-C [Xanthomonas axonopodis pv. citri str. 306]
LysEGL002751	0.3215	lytic endopeptidase preproenzyme [Lysobacter sp. XL1]
LysEGL002784	0.4031	lipase family protein [Cellvibrio japonicus Ueda107]

		LysEGL002811	0.2441	chitinase [Polysphondylium pallidum PN500]
		LysEGL003004	0.187	Heparinase II/III family protein [Desulfatibacillum alkenivorans AK-01]
		LysEGL003254	0.3288	transcription factor jumonji jmjC domain protein [Dyadobacter fermentans DSM 18053]
		LysEGL003255	0.2663	DinB [Bacillus pseudomycoides DSM 12442]
		LysEGL003269	0.3683	putative membrane associated hydrolase [uncultured bacterium BLR18]
		LysEGL003114	0.3271	putative phospholipid membrane-attached protein [Stenotrophomonas maltophilia K279a]
		LysEGL003499	0.2326	putative secreted protein [Xanthomonas campestris pv. musacearum NCPPB4381]
		LysEGL003788	0.3957	bacteriocin-C [Xanthomonas perforans]
		LysEGL003042	0.3895	ATPase [Delftia acidovorans SPH-1]
		LysEGL004365	0.1317	globin [Thioalkalivibrio sp. K90mix]
		LysEGL004577	0.3347	putative hydroxylase [Xanthomonas campestris pv. campestris str. B100]
		LysEGL005114	0.3463	phosphatidate cytidiltransferase [Xanthomonas axonopodis pv. citri str. 306]
V (Cell cycle division, chromosome partitioning)	96h (7)	LysEGL001109	2.2528	cell division protein FtsX [Xanthomonas campestris pv. vesicatoria str. 85-10]
		LysEGL001500	0.3848	rod shape-determining protein [Xanthomonas campestris pv. vasculorum NCPPB702]
		LysEGL001782	0.2551	probable cell division protein ftsi [Xanthomonas albilineans]
		LysEGL001786	0.2481	cell division protein [Xanthomonas campestris pv. campestris str. ATCC 33913]
		LysEGL001790	0.2215	cell division protein [Xanthomonas campestris pv. vasculorum NCPPB702]
		LysEGL001791	0.335	probable cell division protein ftsa [Xanthomonas albilineans]
		LysEGL001792	0.4	cell division protein FtsZ [Stenotrophomonas maltophilia R551-3]
VI (Translation, ribosomal structure and biogenesis)	96h (19)	LysEGL000247	2.1727	putative methionyl-tRNA formyltransferase [Stenotrophomonas maltophilia K279a]
		LysEGL000341	2.3914	tyrosyl-tRNA synthetase [Stenotrophomonas sp. SKA14]
		LysEGL000398	4.3366	pseudouridylate synthase [Xanthomonas campestris pv. campestris str. B100]
		LysEGL001171	2.5532	putative translation factor (sua5) protein [Xanthomonas albilineans]
		LysEGL001812	2.6088	probable pseudouridine synthase protein [Xanthomonas albilineans]
		LysEGL001989	6.5645	Protein methyltransferase HemK [Xanthomonas campestris pv. campestris str. B100]
		LysEGL002237	2.5023	tRNA-i(6)A37 thiotransferase enzyme MiaB [Stenotrophomonas sp. SKA14]

		LysEGL002343	4.1581	tRNA/rRNA methyltransferase [Xylella fastidiosa 9a5c]
		LysEGL003473	2.5746	putative initiation_factor_2b_alpha/beta/delta protein [Xanthomonas albilineans]
		LysEGL003677	3.3862	probable ribonuclease e protein [Xanthomonas albilineans]
		LysEGL004815	5.5854	RNA methyltransferase, TrmH family, group 3 [Stenotrophomonas maltophilia R551-3]
		LysEGL004816	2.24	putative acetyltransferase [Stenotrophomonas maltophilia K279a]
		LysEGL004848	2.3042	tRNA-dihydrouridine synthase A [Stenotrophomonas sp. SKA14]
		LysEGL005209	2.5356	ribosomal RNA small subunit methyltransferase C [Xanthomonas oryzae pv. oryzicola BLS256]
		LysEGL002094	0.4968	ribosomal protein L29 [Stenotrophomonas maltophilia R551-3]
		LysEGL002111	0.3857	probable 50s ribosomal protein l17 [Xanthomonas albilineans]
		LysEGL002280	0.4665	phenylalanyl-tRNA synthetase subunit alpha [Xanthomonas oryzae pv. oryzicola BLS256]
		LysEGL002525	0.4339	probable elongation factor ts (ef-ts) protein [Xanthomonas albilineans]
		LysEGL003979	0.4065	prolyl-tRNA synthetase [Rhodopseudomonas palustris BisA53]
VII (Replication, recombination and repair)	96h (12)	LysEGL000461	4.7632	probable formamidopyrimidine-dna glycosylase protein [Xanthomonas albilineans]
		LysEGL001156	2.5145	putative primosome assembly protein, superfamily II helicase [Xanthomonas albilineans]
		LysEGL001731	2.5934	tyrosine recombinase XerD [Stenotrophomonas sp. SKA14]
		LysEGL002155	2.0852	probable single-stranded dna-binding protein. [Xanthomonas albilineans]
		LysEGL002484	4.6846	ribonuclease HII [Xanthomonas oryzae pv. oryzae MAFF 311018]
		LysEGL004106	3.3122	DNA repair protein RecO [Xanthomonas campestris pv. vesicatoria str. 85-10]
		LysEGL004475	8.4792	DNA methylation and regulatory protein (methylated-DNA--[protein]-cysteine S-methyltransferase) [Xanthomonas campestris pv. campestris str. B100]
		LysEGL004476	5.0099	6-O-methylguanine-DNA methyltransferase [Xanthomonas campestris pv. campestris str. ATCC 33913]
		LysEGL004646	2.8573	Holliday junction resolvase-like protein [Xanthomonas campestris pv. campestris str. ATCC 33913]
		LysEGL002162	0.4377	excinuclease ABC, A subunit [Burkholderia phytofirmans PsJN]

		LysEGL004045	0.3633	probable dna topoisomerase iv subunit a protein [Xanthomonas albilineans]
		LysEGL005227	0.3852	ribonucleoside-diphosphate reductase, adenosylcobalamin-dependent [Comamonas testosteroni KF-1]
VIII (hypothetical protein)	24h (6)	LysEGL003006	3.5739	
		LysEGL003008	2.8103	
		LysEGL003009	3.901	
		LysEGL004723	0.496	
		LysEGL005221	0.331	
		LysEGL005222	0.3243	
	48h (20)	LysEGL000766	2.7538	
		LysEGL000768	2.1961	
		LysEGL001190	2.116	
		LysEGL003233	5.7147	
		LysEGL003822	2.2112	
		LysEGL000121	0.4124	
		LysEGL000524	0.2999	
		LysEGL000784	0.3873	
		LysEGL002959	0.4324	
		LysEGL003151	0.2422	
		LysEGL001849	0.4647	
		LysEGL002960	0.4125	
		LysEGL003610	0.4445	
		LysEGL000907	0.4654	
		LysEGL003519	0.3234	
		LysEGL003847	0.4495	
		LysEGL004553	0.4601	

	LysEGL005219	0.4758
	LysEGL005221	0.415
	LysEGL005222	0.4165
96h (178)	LysEGL000006	2.6162
	LysEGL000011	2.4025
	LysEGL000022	4.0243
	LysEGL000244	2.1512
	LysEGL000239	5.0712
	LysEGL000314	3.9617
	LysEGL000402	2.6948
	LysEGL000420	2.2205
	LysEGL000421	8.0214
	LysEGL000423	3.1626
	LysEGL000651	4.3453
	LysEGL000326	5.8022
	LysEGL000384	3.2474
	LysEGL000520	4.5745
	LysEGL000526	2.2081
	LysEGL000577	5.9761
	LysEGL000784	4.7785
	LysEGL000788	2.608
	LysEGL000884	2.1339
	LysEGL000955	2.4572
	LysEGL000984	4.4546
	LysEGL001167	2.3205
	LysEGL001168	3.2055

LysEGL001199	2.0266
LysEGL001277	2.5946
LysEGL001386	3.1169
LysEGL001432	6.4954
LysEGL001437	2.9882
LysEGL001528	2.6337
LysEGL001715	2.6574
LysEGL001813	5.655
LysEGL002572	4.3519
LysEGL002574	3.0483
LysEGL002877	3.5122
LysEGL002933	3.7732
LysEGL002961	2.8575
LysEGL003439	2.4067
LysEGL003519	6.6096
LysEGL003637	2.423
LysEGL003680	7.3196
LysEGL001548	2.4554
LysEGL001554	2.2741
LysEGL001701	2.1838
LysEGL001721	11.2392
LysEGL001851	2.8896
LysEGL001888	5.9625
LysEGL001977	3.1255
LysEGL002019	2.8726
LysEGL002252	2.174

LysEGL002254	3.3468
LysEGL002317	5.055
LysEGL002483	5.5668
LysEGL002522	2.1613
LysEGL002673	6.8656
LysEGL002696	2.9751
LysEGL002721	2.448
LysEGL002723	2.0042
LysEGL002724	2.1395
LysEGL002763	3.0582
LysEGL002768	4.6638
LysEGL002907	2.4202
LysEGL003054	6.0483
LysEGL003057	2.43
LysEGL003060	2.2826
LysEGL003150	3.0739
LysEGL003151	2.7647
LysEGL003160	2.2889
LysEGL001201	5.5572
LysEGL003203	3.6425
LysEGL003233	2.1411
LysEGL003341	2.4657
LysEGL003384	3.1577
LysEGL003426	2.7505
LysEGL003457	2.0859
LysEGL003475	2.0339

LysEGL003492	4.0516
LysEGL003517	3.6457
LysEGL003530	2.3767
LysEGL003566	3.5143
LysEGL003581	2.1553
LysEGL003839	2.1969
LysEGL003865	5.6034
LysEGL003922	12.8941
LysEGL003948	2.8127
LysEGL004004	2.8957
LysEGL004009	2.1782
LysEGL004035	3.4015
LysEGL004054	2.1436
LysEGL004071	2.0621
LysEGL004121	2.5982
LysEGL004135	2.0792
LysEGL004166	6.1254
LysEGL004168	2.1716
LysEGL004179	5.4239
LysEGL004283	2.0959
LysEGL004351	4.6172
LysEGL004839	3.0662
LysEGL004985	3.2217
LysEGL004429	3.9906
LysEGL004445	6.7975
LysEGL004448	3.2133

LysEGL004566	2.1905
LysEGL004607	3.1146
LysEGL004608	4.2316
LysEGL004660	2.1342
LysEGL004681	4.8421
LysEGL004697	2.0866
LysEGL004717	3.9941
LysEGL004742	3.434
LysEGL004786	2.0582
LysEGL004941	2.6231
LysEGL004951	3.0806
LysEGL005017	2.0547
LysEGL005077	6.6297
LysEGL004975	2.6466
LysEGL005079	2.6896
LysEGL005144	2.6511
LysEGL005155	2.6273
LysEGL005194	4.1245
LysEGL005201	2.336
LysEGL000033	0.4299
LysEGL000153	0.4051
LysEGL000182	0.466
LysEGL000188	0.3855
LysEGL000216	0.2975
LysEGL000465	0.3153
LysEGL000495	0.4083

LysEGL000512	0.4357
LysEGL000750	0.1197
LysEGL000807	0.3888
LysEGL000889	0.3561
LysEGL000907	0.2514
LysEGL000986	0.4972
LysEGL001074	0.4845
LysEGL001075	0.387
LysEGL001122	0.2759
LysEGL001160	0.4954
LysEGL001190	0.4197
LysEGL001228	0.3937
LysEGL001345	0.4816
LysEGL001573	0.2596
LysEGL001575	0.2174
LysEGL001652	0.4028
LysEGL001802	0.4789
LysEGL001849	0.2411
LysEGL002151	0.3707
LysEGL002194	0.1891
LysEGL002195	0.1563
LysEGL002311	0.4006
LysEGL002942	0.2988
LysEGL002986	0.4738
LysEGL003006	0.1276
LysEGL003007	0.1406

LysEGL003008	0.171
LysEGL003009	0.2261
LysEGL003113	0.4048
LysEGL003117	0.4572
LysEGL003166	0.34
LysEGL003024	0.4563
LysEGL003027	0.2215
LysEGL003275	0.2447
LysEGL003358	0.432
LysEGL003359	0.3192
LysEGL003504	0.4427
LysEGL003610	0.2458
LysEGL004210	0.3142
LysEGL004274	0.3759
LysEGL004431	0.4674
LysEGL004437	0.3045
LysEGL004469	0.4514
LysEGL004532	0.4897
LysEGL004561	0.1158
LysEGL004579	0.4201
LysEGL004672	0.3536
LysEGL004783	0.3071
LysEGL004988	0.364
LysEGL005221	0.265
LysEGL005222	0.1208

IX (Cell wall/membrane/envelope biogenesis)	24h (1)	LysEGL004924	2.2362	OmpW family outer membrane protein [Xanthomonas campestris pv. vesicatoria str. 85-10]
	48h (2)	LysEGL003947	2.1473	N-glycosyltransferase [Xanthomonas campestris pv. vasculorum NCPPB702]
		LysEGL000427	2.4709	methyltransferase GidB [Stenotrophomonas sp. SKA14]
	96h (32)	LysEGL000938	2.6457	putative transferase [Pseudomonas fluorescens SBW25]
		LysEGL002066	3.1847	LolB [uncultured bacterium pTW2]
		LysEGL002231	10.3377	secretion protein HlyD family protein [Stenotrophomonas maltophilia R551-3]
		LysEGL002238	4.6533	putative soluble lytic murein transglycosylase precursor [Xanthomonas campestris pv. vesicatoria str. 85-10]
		LysEGL002485	2.3001	lipid-A-disaccharide synthase [Stenotrophomonas sp. SKA14]
		LysEGL002610	2.0741	outer membrane efflux protein [Xanthomonas campestris pv. vesicatoria str. 85-10]
		LysEGL003662	2.5244	GCN5-related N-acetyltransferase [Stenotrophomonas maltophilia R551-3]
		LysEGL003668	2.4416	membrane fusion protein [Stenotrophomonas sp. SKA14]
		LysEGL003674	2.2365	TonB family protein [Stenotrophomonas maltophilia R551-3]
		LysEGL003704	2.6697	putative tonb_protein [Xanthomonas albilineans]
		LysEGL004171	2.0277	saccharide biosynthesis regulatory protein [Stenotrophomonas sp. SKA14]
		LysEGL000102	0.0982	rare lipoprotein A family protein [Stigmatella aurantiaca DW4/3-1]
		LysEGL000812	0.4328	membrane-fusion protein [Shewanella halifaxensis HAW-EB4]
		LysEGL001322	0.4419	component of acridine efflux pump [Stenotrophomonas sp. SKA14]
		LysEGL001499	0.3538	probable penicillin-binding protein. [Xanthomonas albilineans]
		LysEGL001780	0.3762	putative S-adenosyl-methyltransferase mraW [Stenotrophomonas maltophilia K279a]
		LysEGL001785	0.2451	phospho-N-acetylmuramoyl-pentapeptide-transferase [Stenotrophomonas sp. SKA14]
		LysEGL001789	0.2857	putative D-alanine--D-alanine ligase B [Stenotrophomonas maltophilia K279a]
		LysEGL002401	0.4513	putative glycine betaine transporter 2 [Stenotrophomonas maltophilia K279a]
		LysEGL002412	0.4804	wall associated protein [Xanthomonas axonopodis pv. citri str. 306]
		LysEGL002642	0.4253	gp19.3 [Bacillus phage SPO1]

		LysEGL003003	0.2004	glycosyl transferase [Vibrio harveyi ATCC BAA-1116]
		LysEGL003011	0.1397	UDP-N-acetyl-D-mannosamine dehydrogenase [Actinobacillus minor NM305]
		LysEGL003012	0.2245	UDP-N-acetylglucosamine 2-epimerase [Pseudomonas putida GB-1]
		LysEGL003013	0.2395	tyrosine-protein kinase [Pseudomonas fluorescens SBW25]
		LysEGL003016	0.4703	capsular polysaccharide biosynthesis protein [Pseudomonas stutzeri A1501]
		LysEGL003649	0.4494	UTP-glucose-1-phosphate uridylyltransferase [Stenotrophomonas maltophilia R551-3]
		LysEGL003650	0.4889	putative sugar epimerase protein [Xanthomonas albilineans]
		LysEGL004023	0.4621	alginate O-acetylation protein AlgI [Rhodobacterales bacterium Y4I]
		LysEGL004322	0.3135	MltA-interacting protein [Pseudomonas syringae pv. tomato T1]
		LysEGL004924	0.2816	OmpW family outer membrane protein [Xanthomonas campestris pv. vesicatoria str. 85-10]
		LysEGL005043	0.3639	beta-lytic protease [Lysobacter sp. IB-9374]
X (Defense mechanisms)	24h (4)	LysEGL003010	2.4973	polysaccharide biosynthesis protein [Prevotella melaninogenica ATCC 25845]
		LysEGL002232	0.3884	ABC transporter related [Stenotrophomonas maltophilia R551-3]
		LysEGL002233	0.3455	putative ABC transport system, transmembrane protein [Stenotrophomonas maltophilia K279a]
		LysEGL000632	0.3196	aminoglycoside/hydroxyurea antibiotic resistance kinase [Pseudomonas fluorescens Pf0-1]
	96h (9)	LysEGL001542	2.0269	Beta-lactamase class C protein [Agrobacterium radiobacter K84]
		LysEGL005141	2.0652	Uvs125 [uncultured bacterium]
		LysEGL000632	0.4456	aminoglycoside/hydroxyurea antibiotic resistance kinase [Pseudomonas fluorescens Pf0-1]
		LysEGL001313	0.4504	RecName: Full=N-acetylmuramoyl-L-alanine amidase A
		LysEGL001323	0.4962	aminoglycoside/multidrug efflux pump [Herminiimonas arsenicoxydans]
		LysEGL002172	0.4616	YvcC [Bacillus amyloliquefaciens FZB42]
		LysEGL003010	0.2803	polysaccharide biosynthesis protein [Prevotella melaninogenica ATCC 25845]
		LysEGL003251	0.2966	putative transmembrane protein [Stenotrophomonas maltophilia K279a]
		LysEGL005064	0.2647	RND transporter, HAE1/HME family, permease protein [Stenotrophomonas sp. SKA14]

XI (Posttranslational modification)	48h (5)	LysEGL000187	2.0337	Glutathione S-transferase domain [Rhizobium leguminosarum bv. trifolii WSM2304]
		LysEGL001470	0.4588	ATP-dependent protease ATP-binding subunit [Xanthomonas axonopodis pv. citri str. 306]
		LysEGL001962	0.3603	methionine-R-sulfoxide reductase [Sphingomonas wittichii RW1]
		LysEGL001981	0.3876	methionine sulfoxide reductase A [Xanthomonas campestris pv. campestris str. ATCC 33913]
		LysEGL004012	0.4048	heat shock protein HtpX [Xanthomonas axonopodis pv. citri str. 306]
	96h (21)	LysEGL000002	3.7455	putative FAD-dependent pyridine nucleotide-disulphide oxidoreductase [Methylobacterium extorquens AM1]
		LysEGL000294	5.084	O-sialoglycoprotein endopeptidase [Xanthomonas axonopodis pv. citri str. 306]
		LysEGL000375	4.3745	ComM [Xanthomonas oryzae pv. oryzae PXO99A]
		LysEGL001202	2.1266	ADP-ribosylation/Crystallin J1 [Variovorax paradoxus S110]
		LysEGL001904	3.1592	glycoprotease family protein [Stenotrophomonas sp. SKA14]
		LysEGL002734	4.8116	serine protease [Xanthomonas oryzae pv. oryzicola BLS256]
		LysEGL002876	3.3933	FKBP-type peptidyl-prolyl cis-trans isomerase [Xanthomonas oryzae pv. oryzicola BLS256]
		LysEGL002984	2.122	probable chaperone protein dnaj [Xanthomonas albilineans]
		LysEGL003062	2.5789	probable atp-dependent clp protease atp-binding subunit clpa protein [Xanthomonas albilineans]
		LysEGL003067	4.6799	probable leucyl/phenylalanyl-trna--protein transferase (leucyltransferase) [Xanthomonas albilineans]
		LysEGL003552	2.3032	cytochrome c biogenesis protein CcmA [Xanthomonas campestris pv. campestris str. ATCC 33913]
		LysEGL004709	2.1613	heat shock protein [Xanthomonas campestris pv. campestris str. ATCC 33913]
		LysEGL000437	0.3391	PKD repeat-containing protein [Hahella chejuensis KCTC 2396]
		LysEGL000438	0.1284	PKD repeat protein [Pseudoalteromonas tunicata D2]
		LysEGL000570	0.4297	peptidase S8 and S53 subtilisin kexin sedolisin [Stenotrophomonas maltophilia R551-3]
		LysEGL000572	0.478	microbial serine proteinase [Stenotrophomonas sp. SKA14]
		LysEGL000899	0.1538	subtilisin-like protease [Lysobacter sp. IB-9374]
		LysEGL000958	0.3942	putative periplasmic peptidyl-prolyl cis-trans isomerase [Mariprofundus ferrooxydans PV-1]

		LysEGL001641	0.4998	Dna-J like membrane chaperone protein [Stenotrophomonas sp. SKA14]
		LysEGL001866	0.4926	putative organic radical activating enzyme protein [Xanthomonas albilineans]
		LysEGL002819	0.1531	peptidase S8 and S53 subtilisin kexin sedolisin [Herpetosiphon aurantiacus ATCC 23779]
XII (Function unknown and no hit)	24 (4)	LysEGL003026	2.827	
		LysEGL004361	0.4121	
		LysEGL004918	0.3603	
		LysEGL005225	0.4782	
	48h (17)	LysEGL000217	2.3147	
		LysEGL002605	2.6681	
		LysEGL002606	2.3923	
		LysEGL002745	2.5351	
		LysEGL003182	2.0558	
		LysEGL003856	2.2795	
		LysEGL005166	2.3022	
		LysEGL000801	0.3852	
		LysEGL003077	0.4858	
		LysEGL003111	0.4915	
		LysEGL003180	0.4852	
		LysEGL003466	0.3137	
		LysEGL004050	0.4249	
		LysEGL004087	0.3275	
		LysEGL004090	0.3825	
		LysEGL004830	0.4982	
		LysEGL005225	0.385	
	96h (142)	LysEGL000010	2.0187	
		LysEGL000012	2.2828	

LysEGL000023	2.2831
LysEGL000024	2.975
LysEGL000278	2.166
LysEGL000290	3.0061
LysEGL000393	2.8183
LysEGL000664	3.2945
LysEGL000725	2.2431
LysEGL000801	2.0348
LysEGL000909	2.4898
LysEGL000910	2.5805
LysEGL000934	2.557
LysEGL001043	18.3344
LysEGL001098	2.0264
LysEGL001131	2.0203
LysEGL001288	3.6518
LysEGL001329	2.8546
LysEGL001385	3.751
LysEGL001442	2.0895
LysEGL001452	2.2422
LysEGL001571	3.2548
LysEGL001635	3.3063
LysEGL001637	3.3316
LysEGL001725	2.4939
LysEGL001737	2.3666
LysEGL001836	2.8093
LysEGL001870	2.1443

LysEGL002011	2.4959
LysEGL002395	2.0035
LysEGL002431	4.4415
LysEGL002435	5.3691
LysEGL002792	3.8154
LysEGL002812	2.8382
LysEGL002854	2.2681
LysEGL002970	2.0345
LysEGL002971	2.085
LysEGL003234	6.3185
LysEGL003424	2.1058
LysEGL003442	4.6162
LysEGL003458	2.6689
LysEGL003551	3.9304
LysEGL003571	2.2872
LysEGL003817	3.7057
LysEGL003847	2.0536
LysEGL004050	2.1642
LysEGL004089	7.4701
LysEGL004090	3.3067
LysEGL004245	2.6842
LysEGL004250	2.6028
LysEGL004266	5.8478
LysEGL004309	8.9069
LysEGL004352	2.6016
LysEGL004412	4.1368

LysEGL004472	3.0956
LysEGL004591	5.4229
LysEGL004753	2.6709
LysEGL004976	2.9469
LysEGL005129	2.4356
LysEGL000050	0.4591
LysEGL000052	0.4214
LysEGL000079	0.3741
LysEGL000103	0.3965
LysEGL000131	0.3018
LysEGL000137	0.3395
LysEGL000210	0.2729
LysEGL000217	0.4931
LysEGL000388	0.4826
LysEGL000443	0.1156
LysEGL000457	0.3641
LysEGL000579	0.3261
LysEGL000748	0.4616
LysEGL000849	0.4918
LysEGL000903	0.3846
LysEGL000905	0.2629
LysEGL000931	0.3936
LysEGL001036	0.4773
LysEGL001073	0.4084
LysEGL001126	0.491
LysEGL001147	0.4308

LysEGL001150	0.3003
LysEGL001173	0.4229
LysEGL001195	0.1876
LysEGL001579	0.3528
LysEGL001848	0.286
LysEGL001867	0.3261
LysEGL002036	0.1148
LysEGL002039	0.2856
LysEGL002041	0.1876
LysEGL002114	0.4753
LysEGL002125	0.4218
LysEGL002156	0.481
LysEGL002169	0.1488
LysEGL002192	0.4788
LysEGL002207	0.4538
LysEGL002308	0.4194
LysEGL002595	0.1788
LysEGL002604	0.2334
LysEGL002605	0.3409
LysEGL002606	0.34
LysEGL002650	0.2173
LysEGL002670	0.1149
LysEGL002745	0.3685
LysEGL002824	0.2754
LysEGL003014	0.2337
LysEGL003015	0.3374

LysEGL003026	0.4265
LysEGL003063	0.4136
LysEGL003180	0.1505
LysEGL003262	0.302
LysEGL003274	0.2426
LysEGL003276	0.2855
LysEGL003280	0.2891
LysEGL003355	0.3149
LysEGL003357	0.4246
LysEGL003451	0.3943
LysEGL003466	0.3414
LysEGL003562	0.324
LysEGL003769	0.43
LysEGL003823	0.1618
LysEGL003879	0.4424
LysEGL003882	0.3771
LysEGL003930	0.372
LysEGL004039	0.4482
LysEGL004060	0.2859
LysEGL004063	0.3886
LysEGL004086	0.3331
LysEGL004087	0.3943
LysEGL004118	0.4619
LysEGL004219	0.4233
LysEGL004318	0.1491
LysEGL004367	0.4458

		LysEGL004505	0.2012	
		LysEGL004830	0.1923	
		LysEGL004831	0.4395	
		LysEGL004880	0.3733	
		LysEGL004908	0.468	
		LysEGL004918	0.3502	
		LysEGL005001	0.2602	
		LysEGL005092	0.4295	
		LysEGL005107	0.4253	
		LysEGL005225	0.0989	
XIII （Energy production and conversion）	24h (5)	LysEGL005223	0.3095	cytochrome d ubiquinol oxidase, subunit II [Rhodoferax ferrireducens T118]
		LysEGL005224	0.3998	cytochrome D ubiquinol oxidase, subunit I [Legionella pneumophila str. Corby]
		LysEGL001044	0.3451	putative cytochrome bd ubiquinol oxidase, subunitIprotein [Xanthomonas albilineans]
		LysEGL003152	0.2853	nitric oxide dioxygenase [Caulobacter sp. K31]
		LysEGL003521	0.4768	putative membrane associated dehydrogenase (flavoprotein) [Ralstonia eutropha H16]
	48h (2)	LysEGL005223	0.3939	cytochrome d ubiquinol oxidase, subunit II [Rhodoferax ferrireducens T118]
		LysEGL005224	0.4066	cytochrome D ubiquinol oxidase, subunit I [Legionella pneumophila str. Corby]
	96h (25)	LysEGL000673	2.0885	putative oxidoreductase [Xanthomonas campestris pv. musacearum NCPPB4381]
		LysEGL001022	3.3185	putative aldehyde dehydrogenase protein [Xanthomonas albilineans]
		LysEGL001152	2.8105	NADH dehydrogenase protein [Stenotrophomonas sp. SKA14]
		LysEGL001178	3.0244	phosphoenolpyruvate carboxylase [Xanthomonas campestris pv. vesicatoria str. 85-10]
		LysEGL001300	2.8936	putative oxidoreductase, MocA [Burkholderia xenovorans LB400]
		LysEGL001436	2.1569	aldehyde oxidase and xanthine dehydrogenase molybdopterin binding [Methylotenera mobilis JLW8]
		LysEGL001666	2.3938	glycolate oxidase iron-sulfur subunit [Nitrosomonas europaea ATCC 19718]
		LysEGL001869	2.1832	cytochrome c552 [Ruegeria pomeroyi DSS-3]

		LysEGL002003	3.9412	Pnt [uncultured bacterium pTW3]
		LysEGL002476	7.458	NADH: flavin oxidoreductase/NADH oxidase [Burkholderia ambifaria AMMD]
		LysEGL002875	2.9597	Luciferase-like monooxygenase [Stenotrophomonas maltophilia R551-3]
		LysEGL003055	2.1111	bifunctional aconitate hydratase 2/2-methylisocitrate dehydratase [Cupriavidus taiwanensis]
		LysEGL004392	2.4894	glutamate/aspartate: cation symporter family protein [Xanthomonas campestris pv. vesicatoria str. 85-10]
		LysEGL004419	2.7999	formate dehydrogenase accessory protein [Xanthomonas axonopodis pv. citri str. 306]
		LysEGL004450	3.2273	alcohol dehydrogenase [Pseudomonas mendocina ymp]
		LysEGL004931	2.1573	acetyl-CoA hydrolase/transferase family protein [Stenotrophomonas sp. SKA14]
		LysEGL005195	2.5949	dihydrolipoamide acetyltransferase [Xanthomonas campestris pv. vesicatoria str. 85-10]
		LysEGL001124	0.4907	putative glycosyltransferase protein [Xanthomonas albilineans]
		LysEGL001232	0.4197	citrate transporter [Stenotrophomonas maltophilia K279a]
		LysEGL002653	0.2936	ferredoxin reductase-like protein [Lysobacter enzymogenes]
		LysEGL003257	0.4941	luciferase family protein [Sphingomonas wittichii RW1]
		LysEGL003268	0.2747	CtaG [Cystobacter fuscus]
		LysEGL005063	0.3854	glycerol kinase [Xanthomonas campestris pv. campestris str. ATCC 33913]
		LysEGL005223	0.1493	cytochrome d ubiquinol oxidase, subunit II [Rhodoferax ferrireducens T118]
		LysEGL005224	0.1112	cytochrome D ubiquinol oxidase, subunit I [Legionella pneumophila str. Corby]
XIV (Cell motility)	48h (3)	LysEGL001362	2.1693	surface presentation of antigens (SPOA) protein [Burkholderia ambifaria MEX-5]
		LysEGL001376	2.1039	flagellar L-ring protein [Burkholderia ambifaria MEX-5]
		LysEGL002843	2.7254	hemagglutinin [Xanthomonas campestris pv. campestris str. ATCC 33913]
	96h (5)	LysEGL000576	4.1351	periplasmic pilus chaperone [Cupriavidus taiwanensis]
		LysEGL001748	2.8617	probable general secretion pathway protein l [Xanthomonas albilineans]
		LysEGL004656	2.6863	putative twitching mobility protein [Stenotrophomonas maltophilia K279a]
		LysEGL004946	0.4398	fimbrial assembly protein [Xanthomonas axonopodis pv. citri str. 306]
		LysEGL004947	0.4909	Fimbrial assembly membrane protein [Xanthomonas campestris pv. campestris str. ATCC 33913]

Red colors show genes in *L. enzymogenes* up-regulated in the presence of *P. aphanidermatum*, while green colors indicate genes down-regulated (Fold change≥2 or ≤0.5).
