# Supplementary material for: Transcriptional and Antagonistic Responses of Biocontrol Strain Lysobacter enzymogenes OH11 to the Plant Pathogenic Oomycete Pythium aphanidermatum
Source: Front Microbiol. 2017 Jun 6;8:1025. doi: 10.3389/fmicb.2017.01025 (PMC5459918; doi:10.3389/fmicb.2017.01025)
Supplement: Supplementary file 2 [file DataSheet2.DOCX]

**Transcriptional and antagonistic responses of biocontrol strain *Lysobacter* *enzymogenes* OH11 to the plant pathogenic oomycete *Pythium aphanidermatum***

Yangyang Zhao^a^, Guoliang Qian^b^, Yuan Chen^b^, Liangcheng Du^c^, and Fengquan Liu^a*^

^a^Institute of Plant Protection, Jiangsu Academy of Agricultural Sciences, Nanjing 210014, China

^b^College of Plant Protection, Nanjing Agricultural University, Nanjing 210095, China/Key Laboratory of Integrated Management of Crop Diseases and Pests (Nanjing Agricultural University), Ministry of Education

^c^Department of Chemistry, University of Nebraska-Lincoln, Lincoln, Nebraska 68588, United States

^*^To whom correspondence should be addressed. E-mail: [fqliu20011@sina.com](mailto:fqliu20011@sina.com)

**Supporting information**

**Figure S1**. Organization of *LysEGL005221-* *LysEGL005226* gene cluster in *L. enzymogenes* strain OH11.

**Table S2**. RT-qPCR primers of 10 selected differentially-expressed genes used in this study.

**Table S3**. Comparative analysis of selected gene expression levels along the interaction between *L. enzymogenes* and *P*. *aphanidermatum*


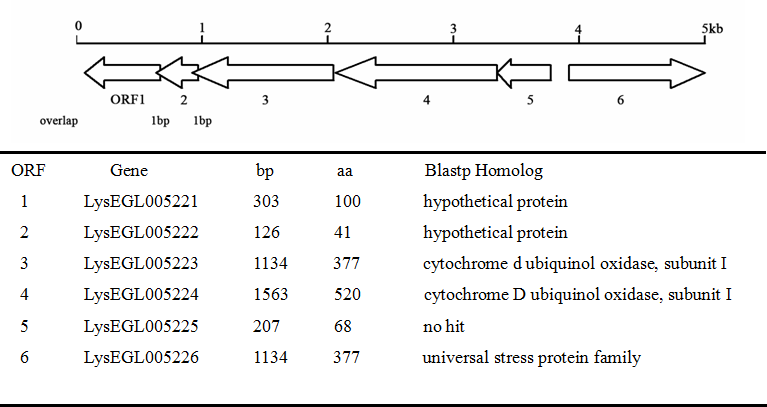


**Figure S1**. Organization of *LysEGL005221-* *LysEGL005226* gene cluster in *L. enzymogenes* strain OH11. These six genes exhibited significantly changed expression levels at all three time points in the interaction.

**Table S2** RT-qPCR primers of 10 selected differentially-expressed genes used in this study

| Locus | Predicted product | Amplification size (bp) | Primer sequence (5′→3′) | Primer name |
| --- | --- | --- | --- | --- |
| LysEGL002651 (*pks-nrps*) | polyketide synthase/nonribosomal peptide synthetase | 116 | TCGAAGTGGCCAAGGAACTG  GAAAGCCTCGAAGTGCTCGTT | PKS-NRPSF  PKS-NRPSR |
| LysEGL002649 (*ox1*) | FAD-dependent ocidoreductase | 127 | CGGTCATCCACGTCACCTACTT  TTGCGCTCCAGGAATTCG | OX1F  OX1R |
| LysEGL002647 (*ox3*) | FAD-dependent ocidoreductase | 234 | CCTACCGCAAGCTCTACACC  AGACGATGCAATGGTCCTTC | OX3F  OX3R |
| LysEGL002646 (*ox4*) | Alcohol dehydrogenase zinc-binding protein | 162 | TTGGGTGGTACGCGAACA  GGCGTGTCCAGGCAGATG | OX4F  OX4R |
| LyEGL000906 | two component system response regulator | 138 | GACGTCCTTCACCAAGATCG  TCCAGGATCACCGGATACAT | 906F  906R |
| LyEGL000979 | two component system transcriptional regulator | 63 | CAACGAAACCCAGATCTTCC  GTCAGGTTCTGCACCGTTTC | 979F  979R |
| LyEGL001610 | two component response regulator | 128 | GGCCATTCTCAGCCTCATC  CATCGTAGCGTTGGGGTTC | 1610F  1610R |
| LyEGL003816 | two component system transcriptional regulator | 144 | GTGCAGGAAGAAGGCAAGG  ATACACCCAGCGGTTGATCT | 3816F  3816R |
| LyEGL002177 | TonB-dependent receptor | 96 | CTACAGCGTCAACTCGGTCA  GTGTAGTCGAGGGTGGTGGT | 2177F  2177R |
| LyEGL003267 | beta-1,3-glucanase A | 94 | CTGCTGACGATCAGCCTGTA  CGGTTTCCTTGTCCATCTTC | 3267F  3267R |

**Table S3** Comparative analysis of selected gene expression levels along the interaction between *L. enzymogenes* and *P*. *aphanidermatum*

|  | | 24 h | | 48 h | | 96 h | |
| --- | --- | --- | --- | --- | --- | --- | --- |
| Functions | Genes | Microarray | RT-qPCR | Microarray | RT-qPCR | Microarray | RT-qPCR |
| Biosynthesis of HSAF | LysEGL002651  (*pks-nrps*) | 3.17 | 9.75±0.99 | 0.55 | 0.24±0.05 | 0.23 | 0.17±0.04 |
|  | LysEGL002649  (*ox1*) | 2.35 | 4.86±0.49 | 0.54 | 0.36±0.13 | 0.19 | 0.17±0.09 |
|  | LysEGL002647  (*ox3*) | 1.55 | 2.30±0.33 | 0.67 | 0.40±0.05 | 0.21 | 0.14±0.09 |
|  | LysEGL002646  (*ox4*) | 1.40 | 1.52±0.16 | 0.62 | 0.42±0.11 | 0.17 | 0.13±0.04 |
| Two-component system | LysEGL000906 | 1.71 | 1.62±0.43 | 0.74 | 0.85±0.10 | 0.38 | 0.46±0.08 |
|  | LysEGL000979 | 0.84 | 1.16±0.16 | 0.87 | 0.56±0.11 | 3.17 | 2.15±0.40 |
|  | LysEGL001610 | 1.02 | 0.73±0.13 | 0.91 | 0.42±0.18 | 2.85 | 1.49±0.18 |
|  | LysEGL003816 | 1.53 | 4.25±0.47 | 1.57 | 2.98±0.62 | 2.32 | 1.91±0.33 |
| TonB-dependent receptor | LysEGL002177 | 1.23 | 0.97±0.12 | 1.05 | 0.84±0.11 | 0.32 | 0.61±0.06 |
| β-1,3-glucanase | LysEGL003267 | 2.17 | 6.46±0.60 | 0.70 | 0.77±0.12 | 0.19 | 0.37±0.05 |
